# Increased levels of circulating MMP3 correlate with severe rejection in face transplantation

**DOI:** 10.1038/s41598-018-33272-7

**Published:** 2018-10-08

**Authors:** Branislav Kollar, Andrey Shubin, Thiago J. Borges, Sotirios Tasigiorgos, Thet Su Win, Christine G. Lian, Simon T. Dillon, Xuesong Gu, Iris Wyrobnik, George F. Murphy, Bohdan Pomahac, Towia A. Libermann, Leonardo V. Riella

**Affiliations:** 1 Division of Plastic Surgery, Department of Surgery, Brigham & Women’s Hospital, Harvard Medical School, Boston, MA 02115 USA; 2 Department of Molecular and Cellular Biology, Harvard University, Cambridge, MA 02135 USA; 3 Schuster Transplantation Research Center, Brigham & Women’s Hospital, Harvard Medical School, Boston, MA 02115 USA; 4 Program in Dermatopathology, Department of Pathology, Brigham & Women’s Hospital, Harvard Medical School, Boston, MA 02115 USA; 5 Beth Israel Deaconess Medical Center Genomics, Proteomics, Bioinformatics and Systems Biology Center, Division of Interdisciplinary Medicine and Biotechnology, Beth Israel Deaconess Medical Center, Harvard Medical School, Boston, MA 02215 USA

**Keywords:** Allotransplantation, Diagnostic markers

## Abstract

Face transplantation is a viable treatment option for carefully selected patients with devastating injuries to the face. However, acute rejection episodes occur in more than 80% of recipients in the first postoperative year. Unfortunately, neither a correlation between histological grades of rejection and anti-rejection treatment nor systemic surrogate markers of rejection in face transplantation are established in clinical routine. Therefore, we utilized next generation aptamer-based SOMAscan proteomics platform for non-invasive rejection biomarker discovery. Longitudinal serum samples from face transplant recipients with long-term follow-up were included in this study. From the 1,310 proteins analyzed by SOMAscan, a 5-protein signature (MMP3, ACY1, IL1R2, SERPINA4, CPB2) was able to discriminate severe rejection from both no-rejection and nonsevere rejection samples. Technical validation on ELISA platform showed high correlation with the SOMAscan data for the MMP3 protein (r_s_ = 0.99). Additionally, MMP3 levels were significantly increased during severe rejection as compared to no-rejection (p = 0.0009) and nonsevere rejection (p = 0.0173) episodes. Pathway analyses revealed significant activation of the metallopeptidase activity during severe face transplant rejection. This pilot study demonstrates the feasibility of SOMAscan to identify non-invasive candidate biomarkers of rejection in face transplantation. Further validation in a larger independent patient cohort is needed.

## Introduction

Face transplantation is a viable reconstructive option for carefully selected patients with severe facial disfigurement^1,2^. Since 2005, 40 such procedures have been performed worldwide^3^. Face transplantation, similar to hand transplantation, belongs to a field called vascularized composite allotransplantation (VCA), which is characterized by the presence of tissues of different function and immunogenicity in the allograft, such as skin, fat, muscle, tendon, bone, bone marrow, nerves and vessels^4^. From these tissues, skin is believed to be the most immunogenic and susceptible for acute rejection^5,6^. In contrast to solid organ transplantation (SOT) in which rejection occurs in about 10–20% of transplant recipients, the incidence of acute rejection in VCA is more than 80% in the first year after transplantation^7–9^. The VCA allografts are available for direct inspection, which is a unique feature compared to SOT. In face transplantation, sentinel flaps may complement the rejection diagnosis and the experience from our center showed good correlation of histological findings between the face allografts and sentinel flaps, so far^10^. However, apart from clinical presentation and biopsy, there are no other assays to diagnose acute rejection in VCA^4^. Therefore, biomarkers to diagnose, monitor or even prevent rejection are of great interest for the field of VCA.

The gold standard to diagnose acute rejection in VCA is the skin biopsy assessed by the Banff classification of skin-containing composite tissues^11^. However, skin biopsy is associated with morbidity to the patient, including scarring, bleeding or infection, hence it is not favorable for frequent monitoring. Furthermore, the Banff grading of skin rejection is semiquantitative, might be prone to intra- and interobserver variability, and lacks a correlation to treatment response^12–15^. Thus, strategies to establish non-invasive monitoring, such as from blood samples, are very desirable for the clinical management as well as to define the best treatment strategy. Since extensive immunosuppression drug administration is associated with increased morbidity to the patient, we sought to investigate whether molecular non-invasive protein markers could potentially help to better discern between different severities of the rejection process.

Only a limited number of studies have evaluated markers associated with acute rejection in face transplantation from peripheral blood samples^16^. As a tool for discovery of biomarkers associated with transplant rejection, the concept of mass spectrometry (MS) based proteomics found in recent years its way into the field of SOT, especially kidney transplantation^17–19^. The SOMAscan proteomics platform, in contrast to MS or immunoassays, uses modified aptamers to bind with high selectivity and affinity to proteins and to quantify their expression levels in biological samples. Due to the dual features of these modified single-stranded oligonucleotides to interact selectively with proteins and to hybridize to the complementary DNA strand, hybridization on a microarray results in a quantitative protein expression read-out^20^. The aptamer-based approach is novel for the field of transplant proteomics. Here we present the first comprehensive evaluation of protein biomarkers from serum samples of 6 face transplant recipients utilizing the SOMAscan technology.

## Results

### Workflow and serum samples

**Figure 1:**
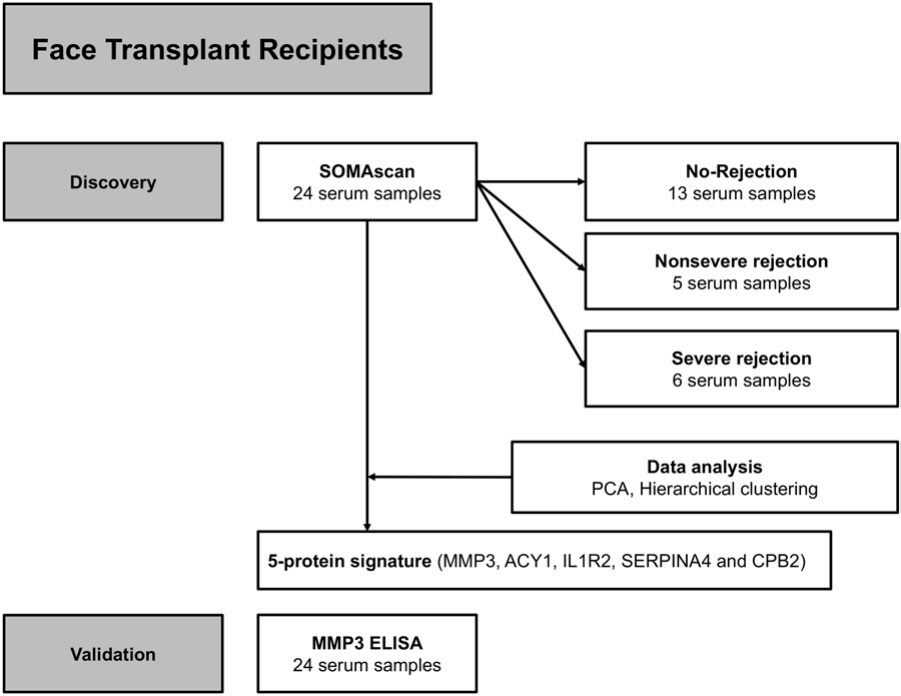
Scheme of the study design. 24 serum samples from all 6 face transplant patients representing no-rejection (n = 13), nonsevere rejection (n = 5) and severe rejection (n = 6) were included into the SOMAscan analysis. Severe rejection episodes required steroid bolus or other more potent drugs for resolution, while nonsevere rejection episodes were reversed by maintenance immunosuppression adjustment and/or topical therapy only. To deem a sample as ‘rejection’, two conditions had to be met simultaneously: Biopsy of grade II or higher and necessity of anti-rejection therapy. Technical validation of SOMAscan data was performed on an ELISA platform.

**Figure 2:**
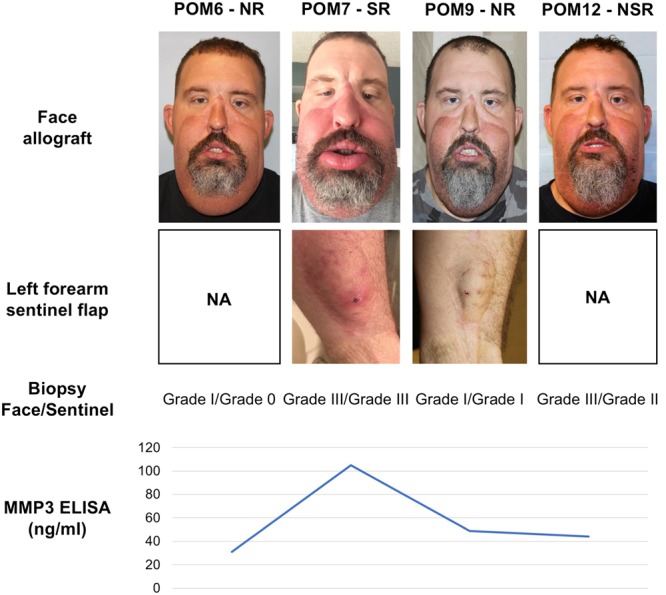
Clinical, histological and non-invasive biomarker findings of a face transplant recipient. The current clinical practice includes assessment of clinical presentation and biopsy findings from both face allograft and sentinel flap as exemplary depicted for patient 5. The patient presented with an acute rejection on POM7 which was marked by facial erythema and edema. The biopsy showed grade III in both face allograft and sentinel flap. The therapy included strong systemic immunosuppression with ATG (severe rejection). The circulating levels of MMP3 were remarkably elevated during this episode. In contrast, the levels of MMP3 were comparable to a no-rejection state at POM12, although the patient had signs of hyperpigmentation in his face as well as grade III and II biopsy results in his face allograft and sentinel flap, respectively. This rejection episode required only topical therapy and adjustment of maintenance immunosuppression for resolution (nonsevere rejection). Clinical photographs of the sentinel flap at POM6 and POM12 were not available. The photograph of the patient at POM7 was previously published by Borges *et al*.^16^ ATG, anti-thymoglobulin; NA, not available; NR, no-rejection; NSR, nonsevere rejection; POM, postoperative month; SR, severe rejection.

A detailed overview of the patients’ characteristics is summarized in Table 1. We retrospectively analyzed management of acute rejection in 6 face transplant recipients whose serum samples were available (Table 2). The data suggested that despite of similar clinical presentation and histological grades of rejection, some acute rejection episodes could be managed by only adjustment of maintenance immunosuppression and/or topical therapy (further referred as ‘nonsevere rejection episodes’), while other acute rejection episodes necessitated intravenous steroid boluses and/or more potent drugs (further referred as ‘severe rejection episodes’) for their resolution. In the first step of the study, a total number of 24 serum samples from all 6 patients with a follow-up range of 12–54 months was included in the proteomic biomarker discovery with SOMAscan: 5 samples represented nonsevere rejection episodes, 6 samples represented severe rejection episodes and 13 samples represented no-rejection episodes. Subsequently, technical validation of SOMAscan results was performed on an ELISA platform (Fig. 1). None of the included rejection samples were from antibody-mediated rejection episodes. To demonstrate the differences between no-rejection, nonsevere rejection and severe rejection states, a set of representative clinical pictures and biopsy findings are shown in Fig. 2.

**Table 1:** Patients’ characteristics.

	Patient 1	Patient 2	Patient 3	Patient 4	Patient 5	Patient 6
Date of transplant	05/2011	03/2011	04/2011	02/2013	03/2014	10/2014
Age at transplant (years)	57	25	30	44	38	33
Gender	F	M	M	F	M	M
Ethnicity	White	White	White	White	White	White
Mechanism of injury	Animal Attack	Electrical Burn	Electrical Burn	Chemical Burn	Ballistic trauma	Ballistic trauma
Facial defect	Missing bilateral eyelids and eyes, nose, maxilla, upper and lower lips	Complete absence of all facial structures	Diffuse scarring, missing nose, parts of upper and lower lips	Diffuse scarring, missing bilateral eyelids, upper and lower lips	Missing nose, maxilla, mandible, upper and lower lips	Missing nose, maxilla, mandible, upper and lower lips
Graft type	Full Face	Full Face	Full Face	Full Face	Partial Face	Partial Face
Bones included into allograft	Nasal, maxilla and zygoma	Nasal	Nasal	Nasal	Nasal, maxilla, zygoma and mandible	Nasal, maxilla, zygoma and mandible
Sentinel flap	Donor’s bilateral upper extremities	Donor radial-forearm flap to recipient’s inguinal area	Donor radial-forearm flap to recipient’s right hand	Donor radial-forearm flap to recipient’s left axilla	Donor radial-forearm flap to recipient’s left forearm	Not available because the face donor was a simultaneous bilateral upper extremity donor
Ischemia time (hours)	2	4	2	3	3	1.5
PRA (%)	0	68	0	97	22	32
DSA	Negative	Negative	Negative	Positive	Negative	Positive
HLA mismatch (A, B, C, DR, DQ, DP)	8	8	5	11	8	7
CMV (Donor/Recipient)	Positive/Positive	Positive/Positive	Positive/Negative	Negative/Positive	Positive/Negative	Negative/Positive
EBV (Donor/Recipient)	Positive/Positive	Positive/Positive	Positive/Positive	Positive/Positive	Positive/Positive	Positive/Positive

The bilateral upper extremities in Patient 1 were removed due to infectious complications in the first postoperative week. DSA, donor specific antibody; PRA, panel reactive antibody.

**Table 2:** Patients’ serum samples.

Patient	Posttransplant month	Histological Banff grade	Status	Clinical presentation	MMP3 ELISA (ng/ml)	Rejection management
Patient 1	12	I	NR	NA	6.04	NA
Patient 1	17	II	NSR	erythema and edema, mucosa lesion	14.37	maintenance immunosuppression adjustment, topical therapy
Patient 1	24	0	NR	NA	7.34	NA
Patient 1	30	III	NSR	subclinical	7.75	maintenance immunosuppression adjustment
Patient 1	42	I	NR	NA	7.3	NA
Patient 2	18	0	NR	NA	11.33	NA
Patient 2	23	II	SR	erythema and edema	70.77	steroid bolus
Patient 2	24	III	SR	erythema and edema	124.6	ATG
Patient 2	48	III	SR	erythema and edema	193.86	steroid bolus
Patient 2	54	I	NR	NA	20.47	NA
Patient 3	12	0	NR	NA	16.66	NA
Patient 3	18	III	SR	exanthema	79.14	steroid bolus
Patient 3	34	III	SR	erythema	24.53	steroid bolus
Patient 3	54	0	NR	NA	20.65	NA
Patient 4	6	I	NR	NA	21.96	NA
Patient 4	13	III	NSR	erythema and edema	18.03	topical therapy
Patient 4	18	0	NR	NA	29.84	NA
Patient 4	24	III	NSR	erythema and edema	39.43	topical therapy
Patient 5	6	I	NR	NA	31.31	NA
Patient 5	7	III	SR	erythema and edema	105.82	steroid bolus, ATG, IVIG
Patient 5	9	I	NR	NA	49.12	NA
Patient 5	12	III	NSR	hyperpigmentation	43.88	maintenance immunosuppression adjustment, topical therapy
Patient 6	3	II	NR	NA	45.92	NA
Patient 6	12	0	NR	NA	23.76	NA

All rejection samples were from cell-mediated rejections. ATG, anti-thymoglobulin; IVIG, intravenous immunoglobulin; NA, not applicable; NR, no-rejection; NSR, nonsevere rejection; SR, severe rejection.

### Signature of 5 serum proteins discriminates best severe rejection from nonsevere and no-rejection episodes

**Figure 3:**
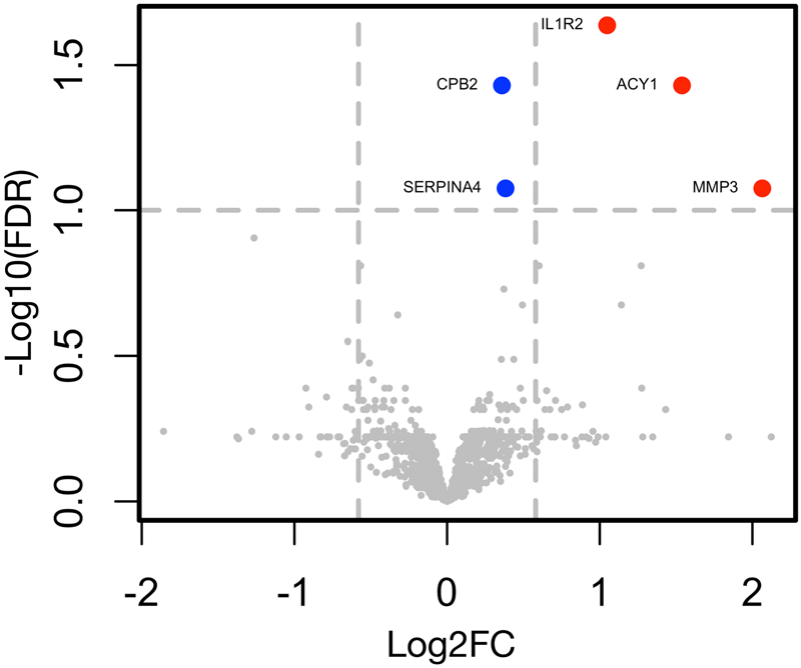
Differential protein expression between the no-rejection and severe rejection episodes. In the volcano plot, the FDR-adjusted p-values (y axis) are compared with log2FC (x axis) in no-rejection (n = 13) versus severe rejection (n = 6) serum samples for all 1310 tested proteins included in the SOMAscan assay. Proteins with FC > 1.5 and FDR < 0.1 are given in red. Proteins with FC < 1.5 and FDR < 0.1 are given in blue. MMP3, ACY1, IL1R2, SERPINA4 and CPB2 were significantly (FDR < 0.1) upregulated during severe rejection episodes as compared to no-rejection episodes. No proteins were found to be significantly downregulated between the severe rejection and no-rejection episodes. FC, fold change; FDR, false discovery rate.

**Figure 4:**
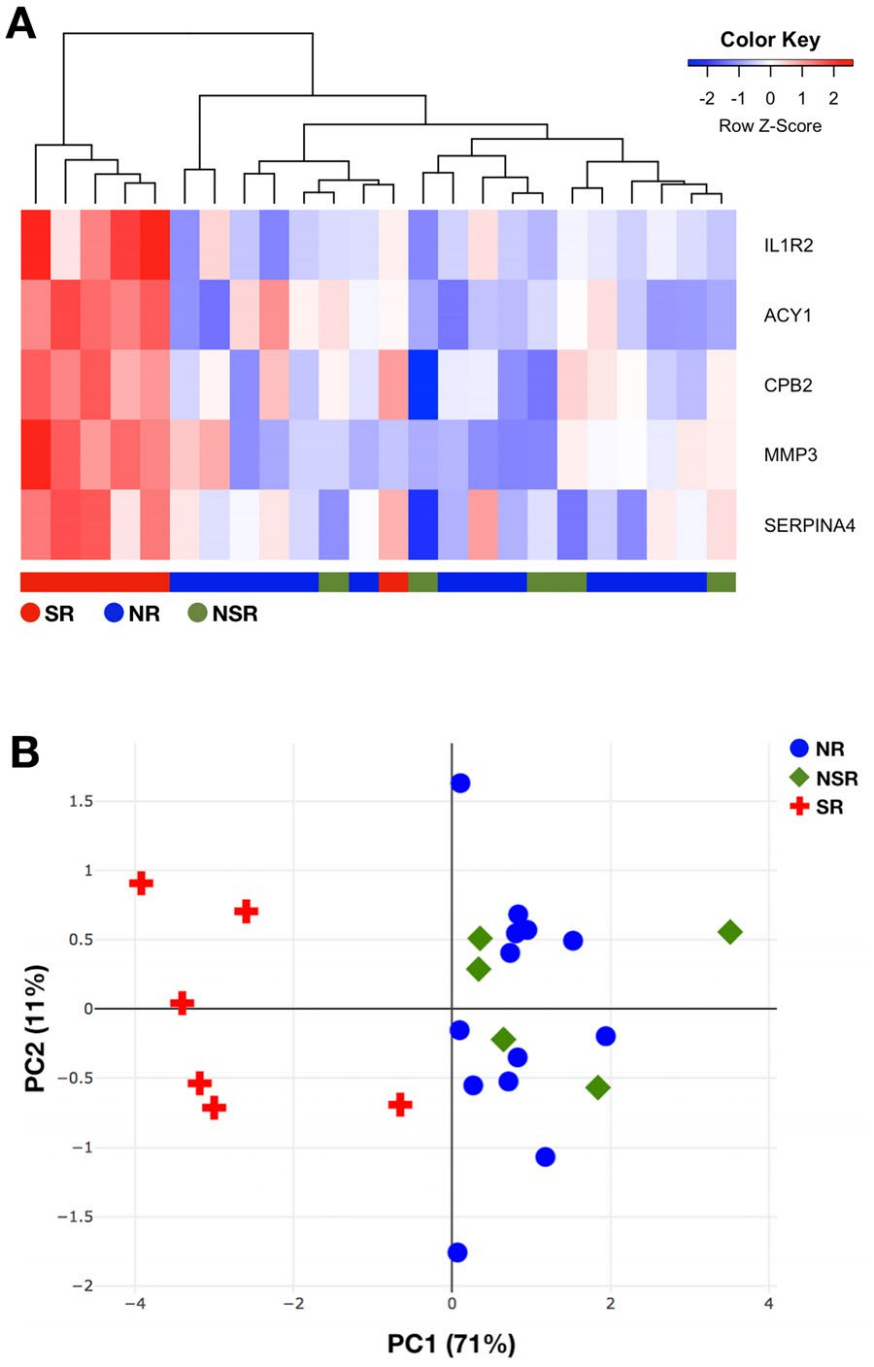
Cluster analysis of the SOMAscan dataset. (**A**) Hierarchical clustering of the five signature proteins could discriminate severe rejection (n = 6) from both nonsevere (n = 5) and no-rejection (n = 13) serum samples. In the heatmap, the rows represent the signature proteins and the columns represent the patients’ samples. To generate the heatmap/cluster dendrogram, Euclidean measure for distance matrix and complete agglomeration method for clustering was applied. (**B**) Principal component analysis based on the five signature proteins could categorize severe rejection samples (red crosses) and no-rejection samples (blue circles) in two distinct clusters separated by the first principal component. Nonsevere rejection samples (green gems) were found in the same cluster as the no-rejection samples. Variance explained by each principal component is given in parentheses. NR, no-rejection; NSR, nonsevere rejection; PC, principal component; SR, severe rejection.

The SOMAscan experiment provided us expression values for 1,310 proteins for every serum sample analyzed. We expected the highest biological variability between the no-rejection and severe rejection states (Fig. 3). Indeed, MMP3 (Matrix Metalloproteinase 3), ACY1 (Aminoacylase-1), IL1R2 (Interleukin-1 receptor type 2), SERPINA4 (Kallistatin) and CPB2 (Carboxypeptidase B2) were found to be significantly upregulated proteins between the no-rejection and severe rejection serum samples (Table 3). MMP3 demonstrated the highest log2 fold change (FC) of 2.07, followed by ACY1 (log2FC = 1.54) and IL1R2 (log2FC = 1.05). The signature of the 5 significantly upregulated proteins was also able to discriminate severe rejection episodes from both no-rejection and nonsevere rejection episodes (Fig. 4A). Comparing severe rejection and nonsevere rejection, at FDR (false discovery rate) threshold of 0.2, two proteins were found to be up-regulated (ACY1 and REN) and one protein downregulated (SHBG) during severe rejection as opposed to nonsevere rejection. No proteins were found to be significantly different between no-rejection and nonsevere rejection episodes, suggesting that nonsevere rejections are not able to trigger systemic response to an extent which could lead to major changes of protein levels measured in blood. This hypothesis can also be made visual by the principal component analysis where every individual sample is represented by a single point in a bidimensional space (Fig. 4B). Analysis based on the 5 signature proteins revealed that no-rejection and severe rejection samples can be found in two distinct clusters, separated by the first principal component. On the other hand, nonsevere rejection samples were found to predominantly cluster with the no-rejection samples (Fig. 4B).

**Table 3:** Significantly up-regulated proteins between ‘severe rejection’ and ‘no-rejection’ groups.

Protein symbol (name)	Log_2_FC	p-value	FDR	Mean (NR)	SD (NR)	Mean (SR)	SD (SR)
MMP3	2.07	3.2e-04	0.084	11.3	0.88	13.38	1.27
ACY1	1.54	8.5e-05	0.037	10.81	0.7	12.35	0.57
IL1R2	1.05	1.8e-05	0.023	12.85	0.27	13.9	0.57
SERPINA4	0.38	2.9e-04	0.084	15.05	0.15	15.43	0.16
CPB2	0.36	6.2e-05	0.037	13.68	0.12	14.04	0.08

Five proteins (MMP3, ACY1, IL1R2, SERPINA4 and CPB2) were demonstrated to be significantly upregulated during severe rejection episodes as compared to no-rejection episodes. MMP3 displayed the highest log2FC, followed by ACY1 and IL1R2. Although significant, SERPINA4 and CPB2 didn’t show biologically relevant FC between the no-rejection and severe rejection groups. The mean/SD values in the table represent the mean/SD of log2 transformed relative fluorescence units from the SOMAscan experiment. FC, fold change; FDR, false discovery rate; NR, no-rejection; SD, standard deviation; SR, severe rejection.

### ELISA for MMP3 is highly correlated with the SOMAscan data and validates enhanced expression of MMP3 during severe acute rejection episodes

**Figure 5:**
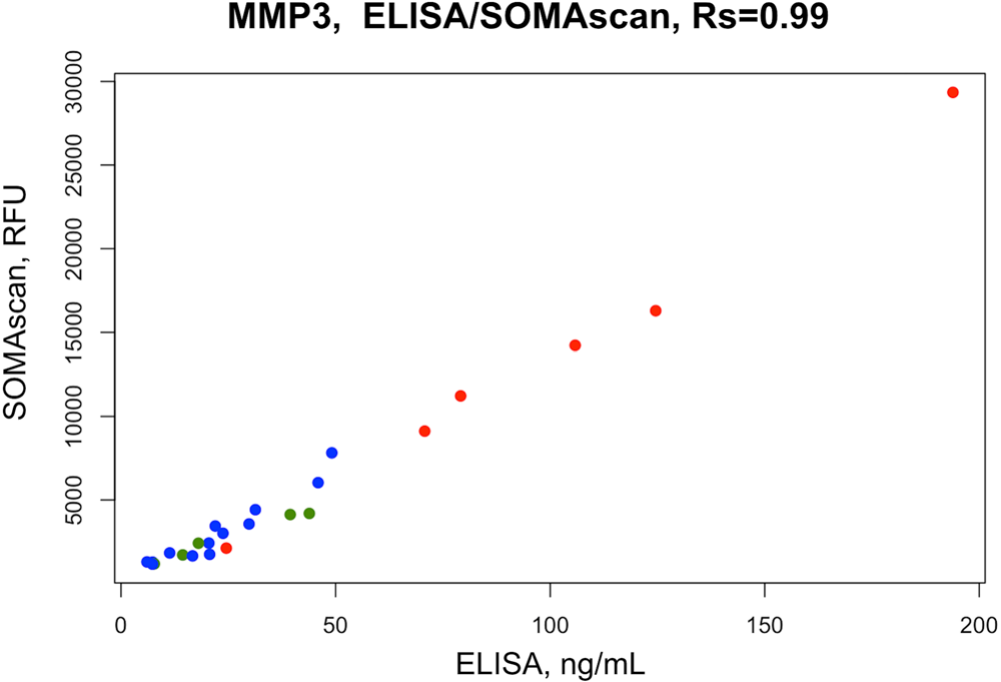
Correlation between SOMAscan and ELISA measurements of MMP3 serum levels. High correlation (Rs = 0.99) was found between SOMAscan and ELISA data for the MMP3 protein, demonstrating that SOMAscan can accurately reflect the relative MMP3 levels in patients’ sera. Severe rejection (n = 6), nonsevere rejection (n = 5) and no-rejection (n = 13) samples are displayed in red, green and blue, respectively. RFU, relative fluorescence units; Rs, Spearman’s rank correlation coefficient.

**Figure 6:**
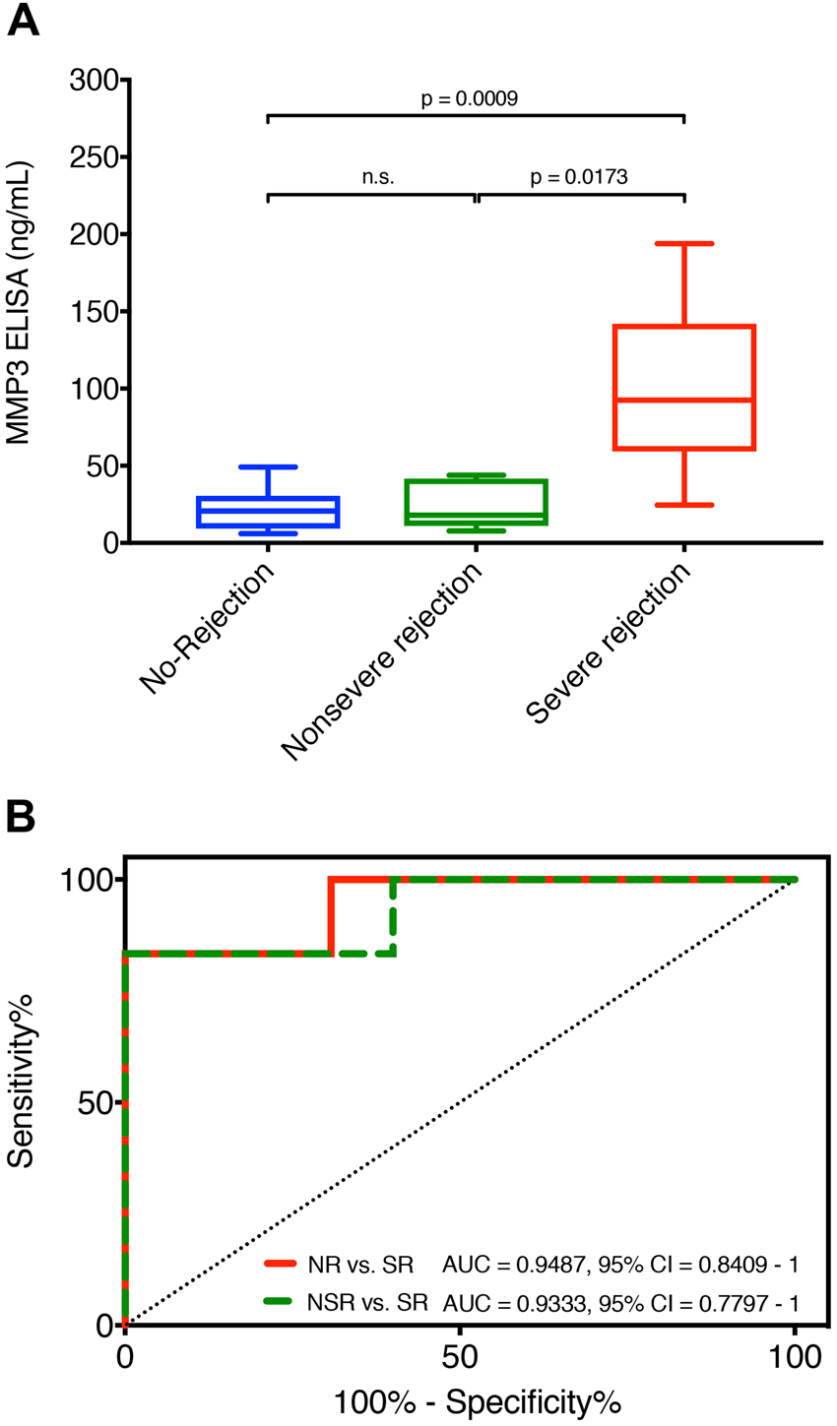
Results of the MMP3 ELISA. (**A**) A total of 24 serum samples from no-rejection (n = 13), nonsevere rejection (n = 5) and severe rejection (n = 6) groups were included in ELISA validation of the SOMAscan results. Serum samples taken at time-points of severe rejections showed significantly higher levels of MMP3 as compared to samples from no-rejection (p = 0.0009) and nonsevere rejection (p = 0.0173) time-points. Data is presented as boxplots: Boxes delineate 1st (lower border) and 3rd (upper border) quartiles from the median (line within the box); whiskers represent minimum and maximum values. Statistical significance was evaluated with a two-tailed non-parametric Mann-Whitney test. (**B**) ROC curves of the MMP3 ELISA data between the no-rejection, nonsevere rejection and severe rejection samples. MMP3 shows good performance as diagnostic marker for severe rejection episodes as compared to no-rejection (AUC = 0.9487; 95% CI 0.8409 to 1) as well as nonsevere rejection (AUC = 0.9333; 95% CI 0.7797 to 1) episodes. n.s., not significant; ROC, receiver operating characteristic.

**Figure 7:**
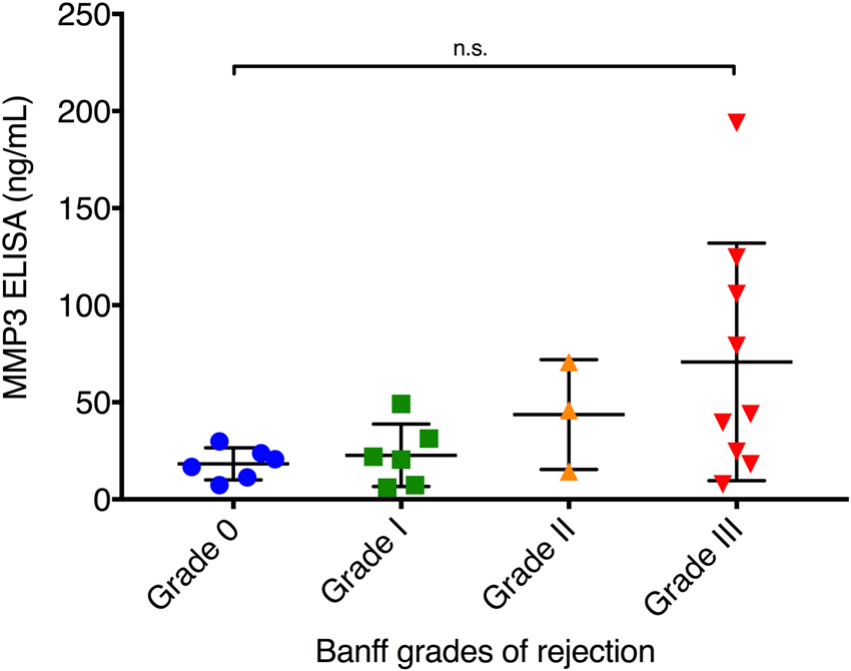
Biopsy adjusted results of the MMP3 ELISA. MMP3 ELISA results from 24 serum samples were stratified according to the histological Banff grades of rejection. A trend towards higher MMP3 levels with increasing Banff grades could be observed, however no statistically significant difference in serum protein levels between any grade of rejection could be demonstrated. Data is presented as scatter dot plot showing every individual value. Mean and standard deviation are displayed as long and short horizontal lines, respectively. Statistical significance was evaluated with a non-parametric Kruskal-Wallis test. n. s., not significant.

To validate the results from the SOMAscan analysis with a gold standard immunoassay technology, we aimed to determine whether the results from the SOMAscan analysis are reproducible with ELISA. We selected MMP3 based on its ranking as the top protein in discriminative signature identified as well as its known role in inflammatory processes and tissue repair (Fig. 1). Indeed, we found high correlation (Spearman’s rank correlation coefficient r_s_ = 0.99) of the SOMAscan and ELISA data for the MMP3 protein (Fig. 5), demonstrating that the SOMAscan data accurately reflect the relative protein expression levels. For better overview, individual MMP3 ELISA values for every included serum sample are also presented in Table 2. MMP3 ELISA further showed that severe rejections displayed significantly higher levels of MMP3 as compared to no-rejection (p = 0.0009) and nonsevere rejection (p = 0.0173) episodes (Fig. 6A). To evaluate the value of MMP3 as a biomarker for transplant rejection, we generated a receiver operating characteristic curve from the ELISA data of the no-rejection and severe rejection serum samples. We found good performance (AUC = 0.9487; 95% CI 0.8409 to 1) for MMP3 as a diagnostic marker of severe rejection episodes (Fig. 6B). Similar performance (AUC = 0.9333; 95% CI 0.7797 to 1) could also be achieved in discrimination of nonsevere from severe rejection episodes (Fig. 6B). In this context, MMP3 levels with cut-off value of 57 ng/mL indicated presence of severe rejection with 83.33% sensitivity and 100% specificity. Since not all patients had a severe rejection event, we excluded samples from patients 1, 4 and 6 who didn’t experience any severe rejections and confirmed the findings above (Supplementary Fig. S1). The MMP3 levels were not notably elevated before the onset of acute rejection in most of the patients (Fig. 2, Table 2). Further, no statistically significant difference in MMP3 levels based on different grades of rejection was demonstrated (Fig. 7), which could support the hypothesis that histological grades alone based on small superficial skin sampling of the graft might not accurately represent the severity of acute rejection.

### Metallopeptidase activity is associated with severe rejection

The advantage of proteomic studies over gene expression studies is that protein synthesis is more likely to represent the molecular phenotype and functional changes than just the gene expression. In order to assess the molecular pathways associated with acute rejection, we performed gene ontology analysis of the five significantly upregulated proteins during severe rejection according to the PANTHER classification system, which identified one significant pathway activation: Metallopeptidase activity (FDR 0.035, Fold Enrichment = 66.1). As expected, MMP3, CBP2 and ACY1 were found to be enriched in this pathway. This result further supports a potential pathophysiological role of MMP3 in rejection process of face allografts.

## Discussion

In the present study, we show that the SOMAscan proteomics platform is able to identify potential biomarkers of face transplant rejection. A signature of 5 proteins could separate severe rejection episodes from both no-rejection and nonsevere rejection episodes. Consecutive technical validation with ELISA showed high data correlation for the MMP3 protein as well as significant increase of MMP3 during severe acute rejection episodes as opposed to nonsevere/no-rejection episodes. SOMAscan has been linked to biomarker discovery of various diseases such as cardiovascular diseases^21,22^, cancer^23–25^, Alzheimer’s disease^26,27^, Duchenne dystrophy^28^ or tuberculosis^29,30^. However, to the best of our knowledge, no published study evaluated its utility for the field of transplantation to date. Most of the approaches of proteomic biomarker discovery in the field of transplantation rely on MS^31,32^. Theoretically, due to normal cell turnaround, any protein of the human body could be present in the blood at minimal concentration^33^. To cover all these proteins, a potential assay should pose a dynamic range of more than 12 logs of concentration^34^. Despite of constant improvements in MS technologies, a trade-off in analyzing complex samples between the comprehensiveness, dynamic range (sensitivity), reproducibility and quantification still exists^35^. On the other hand, immunoassays based on antibody detection provide the advantage of very high sensitivity for protein detection up to attomolar concentrations^36^. However, these assays might be prone to cross-reactivity issues and cannot be highly multiplexed due to a typical dynamic range of only 2–4 logs^37^. The SOMAscan platform aims to fill the gap between the proteomic biomarker discovery platforms by utilizing a modified aptamer-based protein detection approach^20^. According to the manufacturer, SOMAscan is able to assess more than 1,300 proteins simultaneously with sensitivity of 38 fM and dynamic range of more than 8 logs from just 50 µL of biological sample^38^. Taken all together, these advantages led us to evaluate the utility of SOMAscan in face transplantation rejection biomarker discovery as a potential alternative to other proteomic platforms.

We report that severe rejection episodes requiring more extensive therapeutic modalities were mainly associated with higher serum levels of MMP3. MMP3 is a zinc-dependent endopeptidase involved in the breakdown of extracellular matrix in both normal physiological processes and in disease. Presently, not much is known about the role of this protein in transplant rejection of composite allografts. Nonetheless, MMP3 has been previously described as prognostic biomarker in SOT^39,40^. A report from kidney transplantation revealed that patients with chronic transplant nephropathy had significantly higher levels of circulating proMMP3 (inactive precursor of MMP3) as compared to patients with acute rejection, stable graft function or healthy individuals. Further, MMP3 levels were positively correlated with elevated creatinine levels in these patients, indicating a potential pathogenic role of MMP3 in chronic transplant nephropathy^39^. However, it is important to note that in the aforementioned study, the inactive form of MMP3 (proMMP3) was measured. The role of MMP3 has also been studied in context of allogenic hematopoietic cell and lung transplantation. This MS-driven proteomic study with subsequent ELISA verification identified high plasma levels of MMP3 to be associated with bronchiolitis obliterans syndrome post transplantation^40^. Further, similarly to our study, MMP3 appeared to be a diagnostic marker, since the levels of MMP3 were not significantly elevated at time-points before the onset of the pathologic condition^40^. In composite grafts, skin is considered to be the most immunogenic tissue^5^ and extracellular matrix proteins such as collagen or elastic fibers are an essential part of the dermis composition in the skin^41^. Therefore, MMP3 might play a role in the pathophysiological processes taking place in the skin during rejection by cleaving these proteins. However, the role of MMPs goes far beyond that, highlighted by their involvement in signaling and phenotypic changes to cells/tissues, which in turn links them closely to inflammatory conditions, tumor metastasis, vessel remodeling or reparative mechanisms such as wound healing^42^. MMPs, including MMP3, are well known to be expressed by inflammatory cells; however, several studies demonstrated that epithelial cells and fibroblasts are also able of MMPs production, which supports the direct participation of these enzymes in the wound repair and remodeling mechanisms^43–45^. Indeed, MMP3-deficient mice show impaired wound healing due to decreased wound contraction capacity^46^. Taken all together, our results indicate that elevated circulating MMP3 levels during severe acute rejection episodes might be a representation of ongoing tissue damage and repair. These findings let us further hypothesize that such rejection episodes could lead in the long-term to chronic skin changes, such as altered extracellular matrix composition. However, this hypothesis needs to be confirmed with histological studies.

The literature on non-invasive biomarkers in face transplantation or VCA in general is scarce, probably because of the relatively novel nature of these procedures and limited understanding of the immunological responses associated with this kind of transplantation. Most of the biomarkers related to transplant rejection in VCA were evaluated from biopsies in experimental and clinical setting^47–49^. However, skin biopsy is associated with morbidity to the patient such as scarring, risk of infection due to break in skin barrier or even potential rejection triggered through innate immune activation after biopsy injury. Further, histopathologic grading by the Banff classification is semi-quantitative and subject to sampling bias based on the small representative sample that may miss a rejection process by the patchy nature of its presentation^12,50^. Also, the routine 4 mm punch skin biopsy does not assess deeper allograft tissues for rejection. The histological assessment might be additionally prone to intra- and interobserver variability, since the differentiation between “mild” (grade I) and “moderate” (grade II) perivascular inflammatory infiltrate is not objectively defined^15^. Last but not least, the clinical relevance of mild forms of rejection (grade I) by Banff grading is unclear too and justifies the question whether grade I rejections in face transplantation should be treated^51^. In our center, we don’t consider grade I rejection for anti-rejection treatment anymore. We see biopsy as central part of the clinical decision process but not the only criterion to initiate therapy^52^. Therefore, we include in our decision algorithm also other factors such as clinical presentation or medical history (e.g. resolving rejection, exposure to environmental conditions). The potential development of a systemic non-invasive biomarker of severe rejection in face transplantation is clinically promising by allowing to complement the clinical and biopsy data in the decision of treatment options.

All the limitations of the conventional diagnosis of rejection from biopsies are underlined by the fact that no correlation between histological Banff grades and therapy has been established in VCA yet^13,14,53^. Severe rejection episodes are expected to be associated with strong systemic immune response which could be potentially measured in blood circulation. Unfortunately, no such markers of systemic activation are available in VCA to date. Thus, non-invasive biomarkers are of high demand for our field. Studying the same 6 face grafted patients as in the present study, our group found that acute cell-mediated rejection episodes were characterized by increase in IFN-γ/IL-17-producing cells and decrease of FoxP3+ cells in peripheral blood^16^. In the present study we demonstrate that elevated MMP3 levels correlate with severe rejections that required strong systemic immunosuppression. While SOMAscan is not available for biomarker discovery in every center performing face transplantation, the high correlation with ELISA data encourages us to see MMP3 as a potential biomarker, since ELISA can be performed routinely in any hospital. Therefore, this report provides one of the first and important pieces of evidence that molecular non-invasive markers could enhance the diagnostic armamentarium available to clinicians who manage rejection in VCA.

However, our study has several limitations. One of them is that it is difficult to draw conclusions from 6 patients. Indeed, we observed significant variability in the serum reactivity between the patients, which made the data analysis more challenging. Nonetheless, just 40 face transplants have been performed worldwide to date and our patient cohort is one of the largest in the world^3^. Furthermore, face transplantation is the most challenging transplant so far performed with extremely high rejection rates^8^. Therefore, we believe that the evidence drawn from these patients might be relevant for the whole VCA community. The single center nature of this study is also another limitation. It is reasonable to think that in centers with other immunosuppressive strategies and rejection diagnosis/treatment algorithms, the results might be different. Thus, it is important to validate our findings with larger independent patient cohorts in the future. Further studies should also focus on evaluation of molecular patterns of subclinical rejection or other inflammatory skin disorders to make drawing a clear line between these different entities easier for the physicians. Eventually, this might lead to better tailoring of immunosuppressive regimens as well as prevention of acute and chronic rejection.

Overall, face transplantation is now a reality for patients with significant facial deformities and is associated with good mid-term outcomes. However, the high prevalence of rejection urges us to better understand the immune-mediated process and the development of non-invasive biomarkers for the detection of rejection. In this pilot study, we utilize a novel proteomic platform in VCA recipients to detect severe rejection and we hope to extend this initial observation with a multicenter study.

## Methods

### Study approval

All 6 patients gave written informed consent to participate in the clinical trial (ClinicalTrials.gov number, NCT01281267) for face transplantation, as approved by the institutional review board (IRB) at Brigham and Women’s Hospital (Protocol #: 2008BP00055). No organs/tissues were procured from prisoners. The organs were procured following the regulations and guidelines of the New England Organ Bank (NEOB) and all transplants were performed at the Brigham and Women’s Hospital. All patients gave written informed consent to collect and process their blood samples as approved by the IRB at Brigham and Women’s Hospital (Protocol #: 2010P000743). All experiments were performed in accordance with the relevant institutional guidelines and regulations described above. All photographs of patients in this manuscript were produced by the investigators and the patients had provided informed consent for publication of identifying information/images in an online open-access publication.

### Patients and immunosuppression

Six patients were transplanted with facial allografts between March 2011 and October 2014. Donors and recipients were matched according to sex, skin color and ABO compatibility, in addition to a negative T- and B-cell cytotoxic crossmatch. The only exception was a highly sensitized patient with a high panel reactive antibody (98%), in which transplant occurred across a weakly positive cytotoxic T-cell crossmatch (20%)^54^. None of the patients ever received transplants other than VCA. Further patients’ demographic details are given in Table 1.

All patients received mycophenolate mofetil (1,000 mg), methylprednisolone (500 mg), and rabbit anti-thymoglobulin (1.5 mg/kg/day for 4 days) for induction therapy starting at the time of transplant. After transplantation, the maintenance immunosuppression typically consisted of mycophenolate mofetil (1,000 mg twice daily), tacrolimus (adjusted to achieve target levels of 8–12 ng/mL) and prednisone taper^16^. With time, we attempted to withdraw steroids and reduce overall maintenance immunosuppression drug doses if permitted by the clinical and histological graft stability^55^. In case of acute cellular rejection, the rescue therapy was approached by one or a combination of these modalities: steroid bolus (methylprednisolone 500 mg daily for 3 days followed by a taper), adjustment of maintenance immunosuppression or topical therapy. For steroid refractory rejection episodes, anti-thymoglobulin or alemtuzumab were further considered. All acute rejection episodes were successfully managed and no face graft losses, deaths or re-transplantation occurred.

### Diagnosis of rejection

Face allograft biopsies were performed at 3, 6, 12 months and then yearly as well as during suspected rejection. An acute rejection was usually suspected in case of sudden change of graft appearance such as erythema, edema, exanthema or mucosa lesions. This might have been accompanied by pain or overall worsening of the general condition of the patient. Acute rejection was then diagnosed from formalin-fixed, paraffin-embedded 4-mm skin punch biopsies (corresponding to serum samples) in accordance to the Banff classification of skin-containing composite tissues^11^ with following grades: grade 0 = ‘no or rare inflammatory infiltrates’; grade I (mild) = ‘mild perivascular infiltration’ (with no epidermal involvement); grade II (moderate) = ‘moderate-to-severe perivascular inflammation with or without mild epidermal and/or adnexal involvement’; grade III (severe) = ‘dense inflammation and epidermal involvement with epithelial apoptosis, dyskeratosis, and/or keratinolysis’; grade IV (necrotizing acute rejection) = ‘frank necrosis of epidermis or other skin structures’. The biopsy grade was determined as a consensus opinion of at least two independent dermatopathologists (C.G.L. and G.F.M.). A rejection episode was defined as grade II or higher, which required at least one of the treatment modalities described above. The clinical presentation (including sentinel flap appearance), medical history and environmental factors (e.g., exposure to sun) were also taken into account before an anti-rejection treatment was initiated.

### Serum sample collection

Venous blood samples were prospectively collected in Red-Top tubes (no anticoagulant) from transplant recipients at these time-points: pre-transplantation and post-transplantation at 24 hours, 1 week, 3, 6, 12 months, followed by six-monthly intervals; and during suspected rejection. In case of suspected rejection, the samples were taken before therapy was initiated. Serum was isolated from each blood sample and stored at −80 °C in the tissue repository where it could be retrospectively accessed for the proteomic analysis. Once available rejection serum samples were identified, the samples immediately preceding the rejection episodes were selected in order to evaluate the potential predictive value of protein biomarkers.

### Proteomic analysis using the slow-off-rate-modified aptamer SOMAscan platform

SOMAscan analysis (SomaLogic; Boulder, CO) using longitudinal serum samples from six patients with face transplant were performed at the BIDMC Genomics, Proteomics, Bioinformatics and Systems Biology Center. Samples were run using the SOMAscan Assay Kit for human serum, 1.3k (cat. #900–00012), according to the standard protocol for serum from SomaLogic, as described previously^56^. Five pooled serum controls and one no-protein buffer control were run in parallel with the serum samples. Median normalization and calibration of the data was performed according to the standard quality control protocols at SomaLogic. All samples passed the established quality control criteria. A full list of proteins included in the SOMAscan v1.3k assay can be viewed in Supplementary Table S1.

### ELISA validation of SOMAscan

Serum concentrations of MMP-3 from the 6 patients at various time points were measured by enzyme-linked immunosorbent assay (ELISA), using a commercially available kit (R&D Systems cat. #DMP300, Minneapolis, MN), according to the manufacturer’s instructions. All standards and samples were run in duplicate. ELISA plates were read using a BioTek MX plate reader at Optical Density (OD) = 450. A 4-parameter logistic curve was used with final calculations determined in an Microsoft Excel (Redmond, WA) template containing built in macros for optimizing the best-fit model.

### Statistical analysis

Raw SOMAscan data handling, quality-control and log2-transformation for further statistical analysis was carried out with *readat* package^57^. Shapiro-Wilk test was used to check normal distribution of serum levels for each protein in the dataset. Differences in protein serum levels across ‘rejection’ and ‘no-rejection’ groups were calculated via empirical Bayes moderation of the standard errors from linear model fits using *limma* package^58^, as well as using non-parametric pairwise Wilcoxon test. For all multiple comparisons, p-values were adjusted with FDR controlling procedure^59^. Heatmaps and cluster dendrograms were built with *heatmap*.*2* command (Euclidean measure for distance matrix and complete agglomeration method for clustering) within the *gplots* package^60^. Principal component analysis was carried out with *prcomp* function within *stats* package^61^. Plots for principal component analysis were done with *plotly* package^62^. Gene ontology overrepresentation analysis was carried out with PANTHER (version 13.1) using Fisher’s Exact with FDR multiple test correction^63^. Statistical significance of the ELISA data was evaluated with non-parametric two-tailed Mann-Whitney test or Kruskal-Wallis test. All statistical computing was carried out in R statistical environment^61^. The threshold for statistical significance was set as p < 0.05, and for multiple comparisons FDR-adjusted p < 0.1.

## Electronic supplementary material

Supplementary Materials

## Data Availability

The data that support the findings of this study are available from the corresponding author upon reasonable request.
