# Supplementary material for: Increased levels of circulating MMP3 correlate with severe rejection in face transplantation
Source: Sci Rep. 2018 Oct 8;8:14915. doi: 10.1038/s41598-018-33272-7 (PMC6175842; doi:10.1038/s41598-018-33272-7)
Supplement: Supplementary file 1 — Supplementary Materials [file 41598_2018_33272_MOESM1_ESM.pdf]

Supplementary Materials

Increased levels of circulating MMP3 correlate with severe rejection in face transplantation

Branislav Kollar, Andrey Shubin, Thiago J. Borges, Sotirios Tasigiorgos, Thet Su Win, Christine G. Lian, Simon T. Dillon, Xuesong Gu, Iris Wyrobnik, George F. Murphy, Bohdan Pomahac, Towia A. Libermann, Leonardo V. Riella

Table of contents

1. Supplementary Figure S1 Page 2

2. Supplementary Table S1 Page 3 – 26

Supplementary Figure S1

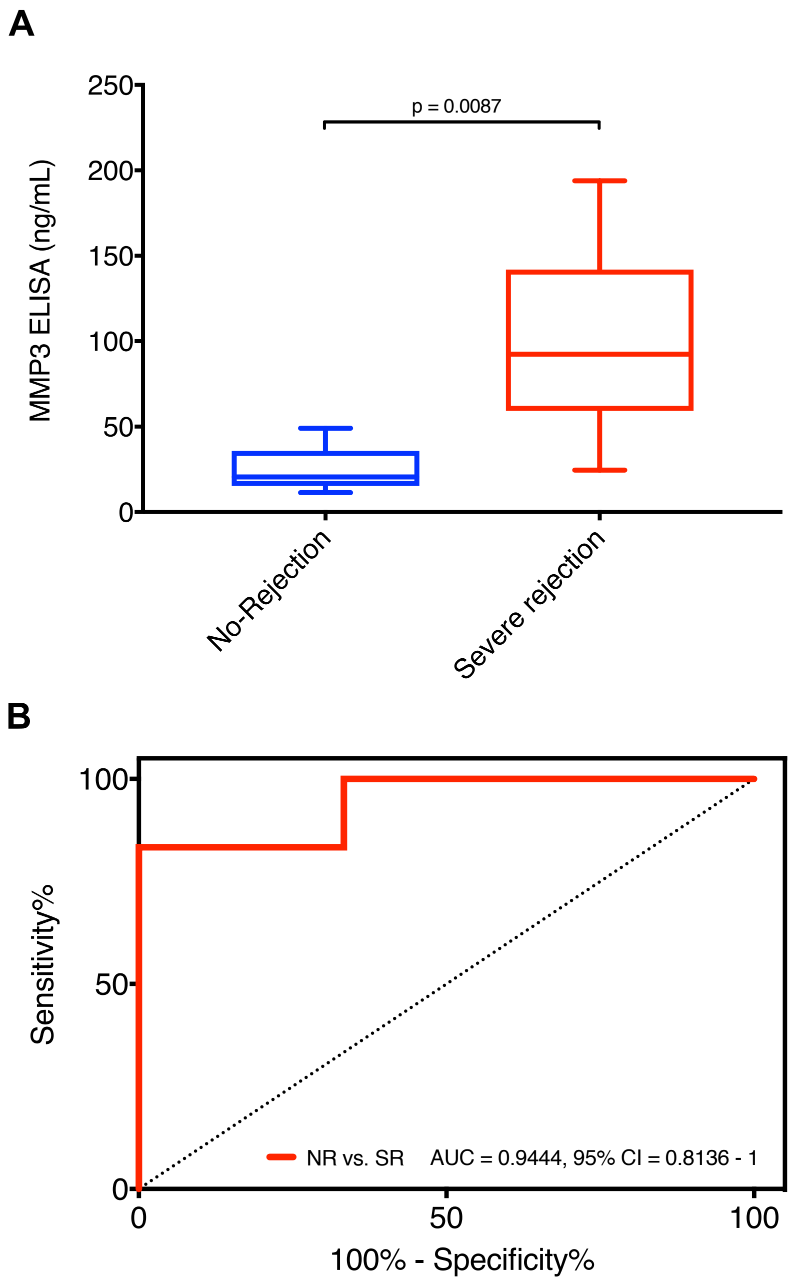

Supplementary Figure S1. Patient adjusted results of the MMP3 ELISA. To avoid confounding by serum samples from patients who didn't have severe rejections, serum samples from patients 1, 4 and 6 were excluded from this analysis. **(A)** During severe rejection episodes, the levels of MMP3 were significantly increased as compared to no-rejection episodes (p=0.0087). Data is presented as boxplots: Boxes delineate 1st (lower border) and 3rd (upper border) quartiles from the median (line within the box); whiskers represent minimum and maximum values. Statistical significance was evaluated with a two-tailed non-parametric Mann-Whitney test. **(B)** ROC curve of the MMP3 ELISA data between the no-rejection (n=6) and severe rejection (n=6) samples. MMP3 shows good performance (AUC= 0.9444; 95% CI 0.8136 to 1) as diagnostic marker for severe rejection episodes. NR, no-rejection; SR, severe rejection; ROC, receiver operating characteristic.

Supplementary Table S1

SOMAscan Assay 1.3k Content

Number	SOMAmer SeqID	Analyte Name	UniProt ID
1	5227-60_3	[Pyruvate dehydrogenase (acetyl-transferring)] kinase isozyme 1, mitochondrial	Q15118
2	14156-33_3	14-3-3 protein beta/alpha	P31946
3	14157-21_3	14-3-3 protein epsilon	P62258
4	4179-57_3	14-3-3 protein family	P31946, P62258, P61981, Q04917, P27348, P63104, P31947
5	4829-43_6	14-3-3 protein sigma	P31947
6	7625-27_3	14-3-3 protein theta	P27348
7	5858-6_5	14-3-3 protein zeta/delta	P63104
8	4995-16_1	15-hydroxyprostaglandin dehydrogenase [NAD(+)]	P15428
9	4563-61_2	1-phosphatidylinositol 4,5-bisphosphate phosphodiesterase gamma-1	P19174
10	10361-25_3	2'-5'-oligoadenylate synthase 1	P00973
11	3898-5_2	26S proteasome non-ATPase regulatory subunit 7	P51665
12	5230-99_3	3-hydroxy-3-methylglutaryl-coenzyme A reductase	P04035
13	4217-49_3	3-hydroxyacyl-CoA dehydrogenase type-2	Q99714
14	5861-78_3	3-hydroxyanthranilate 3,4-dioxygenase	P46952
15	4693-72_2	3-hydroxyisobutyrate dehydrogenase, mitochondrial	P31937
16	4460-8_2	3-phosphoinositide-dependent protein kinase 1	O15530
17	5026-66_1	40S ribosomal protein S3	P23396
18	5484-63_3	40S ribosomal protein S3a	P61247
19	3864-5_2	40S ribosomal protein S7	P62081
20	4957-1_2	40S ribosomal protein SA	P08865
21	2682-68_1	60 kDa heat shock protein, mitochondrial	P10809
22	4187-49_2	6-phosphogluconate dehydrogenase, decarboxylating	P52209
23	4160-49_1	72 kDa type IV collagenase	P08253
24	3174-2_1	A disintegrin and metalloproteinase with thrombospondin motifs 1	Q9UHI8
25	3175-51_5	A disintegrin and metalloproteinase with thrombospondin motifs 13	Q76LX8
26	4533-76_2	A disintegrin and metalloproteinase with thrombospondin motifs 15	Q8TE58
27	2809-25_2	A disintegrin and metalloproteinase with thrombospondin motifs 4	O75173
28	3168-8_2	A disintegrin and metalloproteinase with thrombospondin motifs 5	Q9UNA0
29	5261-13_3	Abelson tyrosine-protein kinase 2	P42684
30	4771-10_3	Acid sphingomyelinase-like phosphodiesterase 3a	Q92484
31	4194-26_3	Acidic leucine-rich nuclear phosphoprotein 32 family member B	Q92688
32	3758-68_3	Activated Protein C	P04070
33	2806-49_2	Activin receptor type-1B	P36896
34	4976-57_1	Adapter molecule crk	P46108
35	5012-67_1	Adenylate kinase isoenzyme 1	P00568
36	5023-23_1	Adenylosuccinate lyase	P30566
37	4546-27_3	Adhesion G protein-coupled receptor E2	Q9UHX3
38	4551-72_3	Adhesion G-protein coupled receptor G5	Q8IZF4
39	3554-24_1	Adiponectin	Q15848
40	11513-92_3	ADP-ribosyl cyclase/cyclic ADP-ribose hydrolase 1	P28907
41	4535-50_2	ADP-ribosyl cyclase/cyclic ADP-ribose hydrolase 2	Q10588
42	14115-34_3	Adrenomedullin	P35318
43	4125-52_2	Advanced glycosylation end product-specific receptor, soluble	Q15109
44	4763-31_3	Afamin	P43652
45	4188-1_2	Aflatoxin B1 aldehyde reductase member 2	O43488
46	3280-49_2	Aggrecan core protein	P16112
47	2813-11_2	Agouti-related protein	O00253
48	3839-60_2	AH receptor-interacting protein	O00170

49	3709-4_2	Alanine aminotransferase 1	P24298
50	4192-10_2	Alcohol dehydrogenase [NADP(+)]	P14550
51	2795-23_3	Alkaline phosphatase, tissue-nonspecific isozyme	P05186
52	2849-49_1	Allograft inflammatory factor 1	P55008
53	4549-78_2	Alpha-(1,3)-fucosyltransferase 5	Q11128
54	2879-9_2	Alpha-1-antichymotrypsin	P01011
55	4153-11_2	Alpha-1-antichymotrypsin complex	P01011
56	3580-25_8	Alpha-1-antitrypsin	P01009
57	3024-18_2	Alpha-2-antiplasmin	P08697
58	3581-53_3	Alpha-2-HS-glycoprotein	P02765
59	3708-62_1	Alpha-2-macroglobulin	P01023
60	3640-14_3	alpha-2-macroglobulin receptor-associated protein	P30533
61	11105-171_3	Alpha-enolase	P06733
62	5792-8_2	alpha-Fetoprotein	P02771
63	3169-70_2	Alpha-L-iduronidase	P35475
64	4292-5_3	Alpha-soluble NSF attachment protein	P54920
65	8458-111_3	Alpha-synuclein	P37840
66	3343-1_4	Aminoacylase-1	Q03154
67	5183-53_3	AMP Kinase (alpha1beta1gamma1)	Q13131 Q9Y478 P54619
68	5245-40_5	AMP Kinase (alpha2beta2gamma1)	P54646 O43741 P54619
69	2970-60_2	Amphiregulin	P15514
70	14134-49_3	Amphoterin-induced protein 2	Q86SJ2
71	3171-57_2	Amyloid beta A4 protein	P05067
72	4874-3_1	Angiogenin	P03950
73	2811-27_1	Angiopoietin-1	Q15389
74	3773-15_4	Angiopoietin-1 receptor, soluble	Q02763
75	2602-2_2	Angiopoietin-2	O15123
76	2500-2_3	Angiopoietin-4	Q9Y264
77	3281-19_1	Angiopoietin-related protein 3	Q9Y5C1
78	3796-79_2	Angiopoietin-related protein 4	Q9BY76
79	3710-49_2	Angiostatin	P00747
80	2805-6_2	Angiotensin-converting enzyme 2	Q9BYF1
81	3484-60_2	Angiotensinogen	P01019
82	7624-19_3	Ankyrin-2	Q01484
83	4960-72_1	Annexin A1	P04083
84	4961-17_1	Annexin A2	P07355
85	14158-17_3	Annexin A5	P08758
86	5335-73_3	Annexin A6	P08133
87	4959-2_1	Anterior gradient protein 2 homolog	O95994
88	4413-3_2	Antileukoproteinase	P03973
89	3344-60_4	Antithrombin-III	P01008
90	2750-3_2	Apolipoprotein A-I	P02647
91	2797-56_2	Apolipoprotein B	P04114
92	4712-28_2	Apolipoprotein D	P05090
93	2418-55_9	Apolipoprotein E	P02649
94	5312-49_3	Apolipoprotein E (isoform E2)	P02649
95	2937-10_2	Apolipoprotein E (isoform E3)	P02649
96	2938-55_2	Apolipoprotein E (isoform E4)	P02649
97	11510-31_3	Apolipoprotein L1	O14791
98	14125-5_3	Apolipoprotein M	O95445
99	3412-7_1	Apoptosis regulator Bcl-2	P10415
100	8447-11_3	Appetite-regulating hormone	Q9UBU3
101	5867-60_3	Arginase-1	P05089
102	3538-26_1	Aromatic-L-amino-acid decarboxylase	P20711

103	2939-10_2	Artemin	Q5T4W7
104	3583-54_4	Arylsulfatase A	P15289
105	3172-28_2	Arylsulfatase B	P15848
106	5452-71_3	Asialoglycoprotein receptor 1	P07306
107	4912-17_1	Aspartate aminotransferase, cytoplasmic	P17174
108	4965-27_1	ATP synthase subunit beta, mitochondrial	P06576
109	9202-309_3	ATP synthase subunit O, mitochondrial	P48047
110	5460-60_3	ATP-dependent RNA helicase DDX19B	Q9UMR2
111	5443-62_2	Atrial natriuretic factor	P01160
112	3875-62_1	AT-rich interactive domain-containing protein 3A	Q99856
113	3091-70_2	Aurora kinase A	O14965
114	3346-72_2	Aurora kinase B	Q96GD4
115	2751-16_2	Azurocidin	P20160
116	4126-22_1	Bactericidal permeability-increasing protein	P17213
117	4973-18_1	Baculoviral IAP repeat-containing protein 3	Q13489
118	3472-40_2	Baculoviral IAP repeat-containing protein 5	O15392
119	4561-65_2	Baculoviral IAP repeat-containing protein 7 Isoform beta	Q96CA5
120	2816-50_2	Basal Cell Adhesion Molecule	P50895
121	3585-54_4	Basigin	P35613
122	13111-79_3	B-cell lymphoma 6 protein	P41182
123	2891-1_3	B-cell receptor CD22	P20273
124	5870-23_2	Bcl2-associated agonist of cell death	Q92934
125	4423-77_2	Bcl-2-like protein 1	Q07817
126	13097-11_3	Bcl-2-like protein 2	Q92843
127	3413-50_2	Bcl-2-related protein A1	Q16548
128	4866-59_2	BDNF/NT-3 growth factors receptor	Q16620
129	3485-28_2	Beta-2-microglobulin	P61769
130	3347-9_2	beta-adrenergic receptor kinase 1	P25098
131	5456-59_2	Beta-Ala-His dipeptidase	Q96KN2
132	13088-397_3	Betacellulin	P35070
133	2558-51_3	Beta-endorphin	P01189
134	5801-72_3	beta-nerve growth factor	P01138
135	5798-3_3	BH3-interacting domain death agonist	P55957
136	3284-75_1	Biglycan	P21810
137	3654-27_4	BMP-binding endothelial regulator protein	Q8N8U9
138	3348-49_2	Bone morphogenetic protein 1	P13497
139	3587-53_2	Bone morphogenetic protein 10	O95393
140	8459-10_3	Bone morphogenetic protein 6	P22004
141	2972-57_2	Bone morphogenetic protein 7	P18075
142	4859-6_1	Bone morphogenetic protein receptor type-1A	P36894
143	4862-63_1	Bone morphogenetic protein receptor type-2	Q13873
144	3415-61_2	Bone sialoprotein 2	P21815
145	3723-1_2	Brain natriuretic peptide 32	P16860
146	2421-7_3	Brain-derived neurotrophic factor	P23560
147	4534-10_2	Brain-specific serine protease 4	Q9GZN4
148	5262-57_3	Breast cancer anti-estrogen resistance protein 3	O75815
149	3461-58_1	Brevican core protein	Q96GW7
150	4328-2_2	Brother of CDO	Q9BWV1
151	4900-8_1	C3a anaphylatoxin	P01024
152	2755-8_2	C3a anaphylatoxin des Arginine	P01024
153	2851-63_3	C5a anaphylatoxin	P01031
154	2501-51_3	Cadherin-1	P12830
155	3656-9_1	Cadherin-12	P55289
156	5410-53_3	Cadherin-15	P55291
157	3797-1_1	Cadherin-2	P19022
158	2643-57_2	Cadherin-3	P22223

159	2819-23_2	Cadherin-5	P33151
160	3591-51_3	Cadherin-6	P55285
161	4903-72_1	Calcineurin	Q08209 P63098
162	3657-74_3	Calcineurin subunit B type 1	P63098
163	5253-1_1	Calcium/calmodulin-dependent 3',5'-cyclic nucleotide phosphodiesterase 1A	P54750
164	3877-67_2	Calcium/calmodulin-dependent protein kinase kinase 1	Q8N5S9
165	3592-4_3	Calcium/calmodulin-dependent protein kinase type 1	Q14012
166	3418-12_2	Calcium/calmodulin-dependent protein kinase type 1D	Q8IU85
167	3350-53_2	Calcium/calmodulin-dependent protein kinase type II subunit alpha	Q9UQM7
168	3351-1_1	Calcium/calmodulin-dependent protein kinase type II subunit beta	Q13554
169	3419-49_2	Calcium/calmodulin-dependent protein kinase type II subunit delta	Q13557
170	2449-1_4	Calcium-dependent phospholipase A2	P39877
171	2668-70_2	Calpain I	P07384 P04632
172	3026-5_2	Calpastatin	P20810
173	5264-65_3	Calreticulin	P27797
174	3466-8_2	cAMP-dependent protein kinase catalytic subunit alpha	P17612
175	4963-19_1	cAMP-regulated phosphoprotein 19	P56211
176	5255-22_3	cAMP-specific 3',5'-cyclic phosphodiesterase 4D	Q08499
177	4469-78_2	Carbohydrate sulfotransferase 15	Q7LFX5
178	4428-1_2	Carbohydrate sulfotransferase 2	Q9Y4C5
179	4429-51_2	Carbohydrate sulfotransferase 6	Q9GZX3
180	4969-2_1	Carbonic anhydrase 1	P00915
181	3420-21_2	Carbonic anhydrase 13	Q8N1Q1
182	4970-55_1	Carbonic anhydrase 2	P00918
183	3799-11_2	Carbonic anhydrase 3	P07451
184	3177-49_2	Carbonic anhydrase 4	P22748
185	3352-80_3	Carbonic anhydrase 6	P23280
186	3356-50_1	Carbonic anhydrase 7	P43166
187	3798-71_1	Carbonic anhydrase 9	Q16790
188	3289-19_2	Carbonic anhydrase-related protein 10	Q9NS85
189	3518-54_2	Carboxypeptidase B2	Q96IY4
190	5343-74_3	Carboxypeptidase E	P16870
191	2889-37_2	Cardiotrophin-1	Q16619
192	5225-50_3	Casein kinase II 2-alpha:2-beta heterotetramer	P68400 P67870
193	5226-36_3	Casein kinase II 2-alpha':2-beta heterotetramer	P19784 P67870
194	3427-63_2	Casein kinase II subunit alpha	P68400
195	5340-24_3	Caspase-10	Q92851
196	4904-7_1	Caspase-2	P42575
197	3593-72_3	Caspase-3	P42574
198	3488-64_2	Catalase	P04040
199	3061-61_2	Cathepsin B	P07858
200	5508-62_3	Cathepsin D	P07339
201	3594-6_5	Cathepsin E	P14091
202	9212-22_3	Cathepsin F	Q9UBX1
203	2431-17_3	Cathepsin G	P08311
204	8465-52_3	Cathepsin H	P09668
205	3364-76_2	Cathepsin L2	O60911
206	3181-50_2	Cathepsin S	P25774
207	4971-1_1	Cathepsin Z	Q9UBR2
208	3676-15_3	Cation-independent mannose-6-phosphate receptor	P11717
209	2770-51_2	C-C motif chemokine 1	P22362
210	4144-13_1	C-C motif chemokine 13	Q99616
211	2900-53_3	C-C motif chemokine 14	Q16627
212	3509-1_1	C-C motif chemokine 15	Q16663
213	4913-78_1	C-C motif chemokine 16	O15467
214	3519-3_2	C-C motif chemokine 17	Q92583

215	3044-3_2	C-C motif chemokine 18	P55774
216	4922-13_1	C-C motif chemokine 19	Q99731
217	2578-67_2	C-C motif chemokine 2	P13500
218	2468-62_3	C-C motif chemokine 20	P78556
219	2516-57_3	C-C motif chemokine 21	O00585
220	3508-78_3	C-C motif chemokine 22	O00626
221	2913-1_2	C-C motif chemokine 23	P55773
222	4128-27_2	C-C motif chemokine 24	O00175
223	2705-5_2	C-C motif chemokine 25	O15444
224	9168-31_3	C-C motif chemokine 26	Q9Y258
225	2192-63_10	C-C motif chemokine 27	Q9Y4X3
226	2890-59_2	C-C motif chemokine 28	Q9NRJ3
227	3040-59_1	C-C motif chemokine 3	P10147
228	2783-18_2	C-C motif chemokine 3-like 1	P16619
229	2781-63_2	C-C motif chemokine 4-like	Q8NHW4
230	5480-49_3	C-C motif chemokine 5	P13501
231	4886-3_1	C-C motif chemokine 7	P80098
232	2785-15_2	C-C motif chemokine 8	P80075
233	10337-83_3	CCAAT/enhancer-binding protein beta	P17676
234	3290-50_2	CD109 antigen	Q6YHK3
235	5451-1_3	CD166 antigen	Q13740
236	13116-25_3	CD177 antigen	Q8N6Q3
237	3029-52_2	CD209 antigen	Q9NNX6
238	5062-60_3	CD226 antigen	Q15762
239	5412-53_3	CD27 antigen	P26842
240	3534-14_2	CD40 ligand	P29965
241	3292-75_1	CD48 antigen	P09326
242	3293-2_4	CD5 antigen-like	O43866
243	11514-196_3	CD59 glycoprotein	P13987
244	9190-7_3	CD63 antigen	P08962
245	5807-77_3	CD70 antigen	P32970
246	5065-8_2	CD83 antigen	Q01151
247	2822-56_2	CD97 antigen	P48960
248	3326-58_2	Cell adhesion molecule 1	Q9BY67
249	3630-27_4	Cell adhesion molecule 3	Q8N126
250	4541-49_2	Cell adhesion molecule-related/down-regulated by oncogenes	Q4KMG0
251	5103-30_3	Cell surface glycoprotein CD200 receptor 1	Q8TD46
252	6152-111_3	Cellular tumor antigen p53	P04637
253	4962-52_1	Cerebral dopamine neurotrophic factor	Q49AH0
254	5246-64_3	cGMP-dependent 3',5'-cyclic phosphodiesterase	O00408
255	5254-69_3	cGMP-inhibited 3',5'-cyclic phosphodiesterase A	Q14432
256	5256-86_3	cGMP-specific 3',5'-cyclic phosphodiesterase	O76074
257	11104-13_3	Chitinase-3-like protein 1	P36222
258	3600-2_3	Chitotriosidase-1	Q13231
259	5013-2_1	Chloride intracellular channel protein 1	O00299
260	13117-232_3	Choline/ethanolamine kinase	Q9Y259
261	3362-61_2	Chordin-like protein 1	Q9BU40
262	13103-125_3	Chorionic somatomammotropin hormone	P0DML2 P0DML3
263	4540-11_2	Chromobox protein homolog 5	P45973
264	8476-11_3	Chromogranin-A	P10645
265	3423-59_2	Chymase	P23946
266	3489-9_2	Ciliary neurotrophic factor	P26441
267	2711-6_2	Ciliary neurotrophic factor receptor subunit alpha	P26992
268	3028-36_2	Ck-beta-8-1	P55773
269	4542-24_2	Clusterin	P10909
270	5066-134_3	CMRF35-like molecule 6	Q08708

271	4905-63_1	Coactosin-like protein	Q14019
272	4876-32_1	Coagulation factor IX	P00740
273	5307-12_3	Coagulation factor IXab	P00740
274	4906-35_1	Coagulation Factor V	P12259
275	3184-25_2	Coagulation factor VII	P08709
276	4878-3_1	Coagulation Factor X	P00742
277	3077-66_2	Coagulation factor Xa	P00742
278	2190-55_1	Coagulation Factor XI	P03951
279	4203-50_2	Cofilin-1	P23528
280	3234-23_2	Coiled-coil domain-containing protein 80	Q76M96
281	4807-13_3	Collagen alpha-1(VIII) chain	P27658
282	4543-65_2	Collagen alpha-1(XXIII) chain	Q86Y22
283	4925-54_2	Collagenase 3	P45452
284	4430-44_3	Collectin-11	Q9BWP8
285	5457-5_2	Collectin-12	Q5KU26
286	2823-7_1	COMM domain-containing protein 7	Q86VX2
287	2753-2_2	Complement C1q subcomponent	P02745 P02746 P02747
288	3285-23_2	Complement C1r subcomponent	P00736
289	3590-8_3	Complement C1s subcomponent	P09871
290	3186-2_2	Complement C2	P06681
291	2754-50_2	Complement C3	P01024
292	4480-59_2	Complement C3b	P01024
293	2683-1_2	Complement C3b, inactivated	P01024
294	5803-24_3	Complement C3d fragment	P01024
295	4481-34_2	Complement C4	P0C0L4, P0C0L5
296	2182-54_1	Complement C4b	P0C0L4 P0C0L5
297	2381-52_4	Complement C5	P01031
298	4482-66_2	Complement C5b-C6 complex	P01031,P13671
299	4967-1_1	Complement component 1 Q subcomponent-binding protein, mitochondrial	Q07021
300	14136-234_3	Complement component C1q receptor	Q9NPY3
301	4127-75_1	Complement component C6	P13671
302	2888-49_2	Complement component C7	P10643
303	2429-27_4	Complement component C8	P07357,P07358,P07360
304	3060-43_2	Complement component C9	P02748
305	5069-9_3	Complement decay-accelerating factor	P08174
306	4129-72_1	Complement factor B	P00751
307	2946-52_2	Complement factor D	P00746
308	4159-130_1	Complement factor H	P08603
309	3666-17_4	Complement factor H-related protein 5	Q9BXR6
310	2567-5_6	Complement factor I	P05156
311	2975-19_2	Connective tissue growth factor	P29279
312	4544-4_3	Connective tissue-activating peptide III	P02775
313	2974-61_2	Contactin-1	Q12860
314	3296-92_2	Contactin-2	Q02246
315	3298-52_2	Contactin-4	Q8IWV2
316	3299-29_2	Contactin-5	O94779
317	5346-24_3	Copine-1	Q99829
318	4785-30_3	Corticosteroid-binding globulin	P08185
319	4890-10_1	Corticotropin	P01189
320	4337-49_2	C-reactive protein	P02741
321	3800-71_2	Creatine kinase B-type	P12277
322	2670-67_4	Creatine kinase M-type	P06732
323	3714-49_2	Creatine kinase M-type:Creatine kinase B-type heterodimer	P12277 P06732
324	3294-55_2	Cryptic protein	P0CG37
325	4332-6_2	C-type lectin domain family 1 member B	Q9P126
326	3361-26_2	C-type lectin domain family 4 member K	Q9UJ71

327	3030-3_2	C-type lectin domain family 4 member M	Q9H2X3
328	3603-60_5	C-type lectin domain family 7 member A	Q9BXN2
329	3041-55_2	C-type mannose receptor 2	Q9UBG0
330	4141-79_1	C-X-C motif chemokine 10	P02778
331	3038-9_2	C-X-C motif chemokine 11	O14625
332	3487-32_2	C-X-C motif chemokine 13	O43927
333	2436-49_4	C-X-C motif chemokine 16	Q9H2A7
334	2979-8_2	C-X-C motif chemokine 5	P42830
335	3495-15_2	C-X-C motif chemokine 6	P80162
336	9188-119_3	C-X-C motif chemokine 9	Q07325
337	3422-4_2	Cyclin-dependent kinase 1:G2/mitotic-specific cyclin-B1 complex	P06493 P14635
338	3357-67_2	Cyclin-dependent kinase 2:Cyclin-A2 complex	P24941 P20248
339	3358-51_2	Cyclin-dependent kinase 5:Cyclin-dependent kinase 5 activator 1 complex	Q00535 Q15078
340	3359-11_2	Cyclin-dependent kinase 8:Cyclin-C complex	P49336 P24863
341	3719-2_2	Cyclin-dependent kinase inhibitor 1B	P46527
342	2609-59_2	Cystatin-C	P01034
343	3803-10_2	Cystatin-D	P28325
344	3302-58_1	Cystatin-F	O76096
345	3303-23_2	Cystatin-M	Q15828
346	3802-50_1	Cystatin-S	P01036
347	4324-33_2	Cystatin-SA	P09228
348	5459-33_3	Cystatin-SN	P01037
349	9171-11_3	Cysteine and glycine-rich protein 3	P50461
350	3187-52_2	Cysteine-rich secretory protein 3	P54108
351	7628-40_3	Cysteine-rich with EGF-like domain protein 1	Q96HD1
352	2942-50_2	Cytochrome c	P99999
353	2943-5_2	Cytochrome P450 3A4	P08684
354	2634-2_2	Cytokine receptor common subunit gamma	P31785
355	2607-54_2	Cytokine receptor-like factor 1:Cardiotrophin-like cytokine factor 1 Complex	O75462 Q9UBD9
356	2746-56_1	Cytokine receptor-like factor 2	Q9HC73
357	5014-49_1	Cytoplasmic protein NCK1	P16333
358	3414-40_2	Cytoplasmic tyrosine-protein kinase BMX	P51813
359	5345-51_3	Cytoskeleton-associated protein 2	Q8WWK9
360	3192-3_2	Cytosolic non-specific dipeptidase	Q96KP4
361	5068-54_3	Cytotoxic and regulatory T-cell molecule	O95727
362	3022-4_2	Cytotoxic T-lymphocyte protein 4	P16410
363	4314-12_2	dCTP pyrophosphatase 1	Q9H773
364	4907-56_1	D-dimer	P02671 P02675 P02679
365	4355-13_1	Death-associated protein kinase 2	Q9UIK4
366	2666-53_2	Decorin	P07585
367	5349-69_3	Delta-like protein 1	O00548
368	3305-6_1	Delta-like protein 4	Q9NR61
369	4979-34_2	Dermatopontin	Q07507
370	4389-2_1	Desert hedgehog protein N-product	O43323
371	13126-52_3	Desmocollin-2	Q02487
372	4981-6_1	Desmocollin-3	Q14574
373	2976-58_2	Desmoglein-1	Q02413
374	5071-3_3	Desmoglein-2	Q14126
375	3122-6_2	Diablo homolog, mitochondrial	Q9NR28
376	3644-5_4	Dickkopf-like protein 1	Q9UK85
377	3535-84_1	Dickkopf-related protein 1	O94907
378	3607-71_1	Dickkopf-related protein 3	Q9UBP4
379	3365-7_2	Dickkopf-related protein 4	Q9UBT3
380	3178-5_2	Dipeptidyl peptidase 1	P53634
381	3608-12_5	Dipeptidyl peptidase 2	Q9UHL4
382	3606-2_5	Discoidin domain-containing receptor 2	Q16832

383	4420-7_2	Disintegrin and metalloproteinase domain-containing protein 12	O43184
384	3795-6_2	Disintegrin and metalloproteinase domain-containing protein 9	Q13443
385	2871-73_2	DNA repair protein RAD51 homolog 1	Q06609
386	2876-74_2	DNA topoisomerase 1	P11387
387	3852-19_2	DnaJ homolog subfamily B member 1	P25685
388	9175-48_3	Down syndrome cell adhesion molecule	O60469
389	4978-54_2	Drebrin-like protein	Q9UJU6
390	5252-33_3	Dual 3',5'-cyclic-AMP and -GMP phosphodiesterase 11A	Q9HCR9
391	2864-2_3	Dual specificity mitogen-activated protein kinase kinase 1	Q02750
392	3628-3_4	Dual specificity mitogen-activated protein kinase kinase 2	P36507
393	6151-18_3	Dual specificity mitogen-activated protein kinase kinase 3	P46734
394	5242-37_3	Dual specificity mitogen-activated protein kinase kinase 4	P45985
395	3480-7_1	Dual specificity protein phosphatase 3	P51452
396	4359-87_1	Dual specificity tyrosine-phosphorylation-regulated kinase 3	O43781
397	5879-51_3	Dynactin subunit 2	Q13561
398	3881-49_2	Dynein light chain 1, cytoplasmic	P63167
399	3845-51_2	Dynein light chain roadblock-type 1	Q9NP97
400	10342-55_3	E3 SUMO-protein ligase PIAS4	Q8N2W9
401	10336-3_3	E3 ubiquitin-protein ligase CHIP	Q9UNE7
402	4245-80_2	E3 ubiquitin-protein ligase Mdm2	Q00987
403	14120-2_3	E3 ubiquitin-protein ligase RNF43	Q68DV7
404	14122-132_3	E3 ubiquitin-protein ligase ZNRF3	Q9ULT6
405	2826-53_2	Ectodysplasin-A, secreted form	Q92838
406	3182-38_2	Ectonucleoside triphosphate diphosphohydrolase 1	P49961
407	4436-1_2	Ectonucleoside triphosphate diphosphohydrolase 3	O75355
408	4437-56_3	Ectonucleoside triphosphate diphosphohydrolase 5	O75356
409	4435-66_2	Ectonucleotide pyrophosphatase/phosphodiesterase family member 7	Q6UWV6
410	8480-29_3	EGF-containing fibulin-like extracellular matrix protein 1	Q12805
411	4982-54_1	Elafin	P19957
412	5882-34_2	Elongation factor 1-beta	P24534
413	4908-6_1	Endoglin	P17813
414	4964-67_1	Endoplasmic reticulum aminopeptidase 1	Q9NZ08
415	4983-6_1	Endoplasmic reticulum resident protein 29	P30040
416	2201-17_6	Endostatin	P39060
417	2981-9_3	Endothelial cell-selective adhesion molecule	Q96AP7
418	3805-16_2	Endothelial cell-specific molecule 1	Q9NQ30
419	2714-78_2	Endothelial monocyte-activating polypeptide 2	Q12904
420	3611-70_4	Endothelin-converting enzyme 1	P42892
421	3189-61_2	Enteropeptidase	P98073
422	5301-7_3	Eotaxin	P51671
423	3431-54_2	Ephrin type-A receptor 1	P21709
424	5076-53_2	Ephrin type-A receptor 10	Q5JZY3
425	4834-61_2	Ephrin type-A receptor 2	P29317
426	3432-21_2	Ephrin type-A receptor 3	P29320
427	3806-55_1	Ephrin type-A receptor 5	P54756
428	5077-28_3	Ephrin type-B receptor 2	P29323
429	3612-6_4	Ephrin type-B receptor 4	P54760
430	5078-82_3	Ephrin type-B receptor 6	O15197
431	14124-6_3	Ephrin-A2	O43921
432	14153-8_3	Ephrin-A3	P52797
433	2614-28_2	Ephrin-A4	P52798
434	2615-60_2	Ephrin-A5	P52803
435	13104-32_3	Ephrin-B1	P98172
436	14131-37_3	Ephrin-B2	P52799
437	2514-65_3	Ephrin-B3	Q15768
438	5509-7_3	Epidermal growth factor	P01133

439	2677-1_1	Epidermal growth factor receptor	P00533
440	4212-5_3	Epidermal growth factor receptor substrate 15-like 1	Q9UBC2
441	5328-33_37	Epidermal growth factor receptor variant III	P00533
442	4956-2_1	Epiregulin	O14944
443	4122-12_2	Epithelial discoidin domain-containing receptor 1	Q08345
444	5813-58_3	Erythropoietin	P01588
445	2715-25_2	Erythropoietin receptor	P19235
446	3470-1_2	E-selectin	P16581
447	4708-3_2	Estradiol 17-beta-dehydrogenase 1	P14061
448	2945-25_1	Estrogen receptor	P03372
449	4997-19_1	Eukaryotic initiation factor 4A-III	P38919
450	4230-1_2	Eukaryotic translation initiation factor 4 gamma 2	P78344
451	4184-43_3	Eukaryotic translation initiation factor 4E-binding protein 2	Q13542
452	5885-55_3	Eukaryotic translation initiation factor 4H	Q15056
453	2612-5_2	Eukaryotic translation initiation factor 5	P55010
454	5888-29_3	Eukaryotic translation initiation factor 5A-1	P63241
455	3366-51_2	Extracellular matrix protein 1	Q16610
456	8463-2_3	Extracellular superoxide dismutase [Cu-Zn]	P08294
457	5032-64_1	FACT complex subunit SSRP1	Q08945
458	4985-11_1	Fatty acid-binding protein, epidermal	Q01469
459	5437-63_3	Fatty acid-binding protein, heart	P05413
460	11516-7_3	Fatty acid-binding protein, liver	P07148
461	4440-15_2	Fc receptor-like protein 3	Q96P31
462	5934-1_3	Ferritin	P02794 P02792
463	3367-8_3	Fetuin-B	Q9UGM5
464	2796-62_2	Fibrinogen	P02671 P02675 P02679
465	4989-7_1	Fibrinogen gamma chain	P02679
466	3486-58_2	Fibroblast growth factor 1	P05230
467	2441-2_4	Fibroblast growth factor 10	O15520
468	4392-54_2	Fibroblast growth factor 12	P61328
469	4393-3_1	Fibroblast growth factor 16	O43320
470	3494-71_3	Fibroblast growth factor 17	O60258
471	2761-49_2	Fibroblast growth factor 18	O76093
472	2762-30_2	Fibroblast growth factor 19	O95750
473	3025-50_1	Fibroblast growth factor 2	P09038
474	2763-66_2	Fibroblast growth factor 20	Q9NP95
475	3807-1_2	Fibroblast growth factor 23	Q9GZV9
476	4123-60_2	Fibroblast growth factor 4	P08620
477	3065-65_1	Fibroblast growth factor 5	P12034
478	4130-71_1	Fibroblast growth factor 6	P10767
479	4487-1_1	Fibroblast growth factor 7	P21781
480	4394-71_2	Fibroblast growth factor 8 isoform A	P55075
481	2443-10_4	Fibroblast growth factor 8 isoform B	P55075
482	2764-20_2	Fibroblast growth factor 9	P31371
483	5532-53_3	Fibroblast growth factor receptor 1	P11362
484	3808-76_2	Fibroblast growth factor receptor 2	P21802
485	3809-1_2	Fibroblast growth factor receptor 3	P22607
486	4988-49_2	Fibroblast growth factor receptor 4	P22455
487	4131-72_2	Fibronectin	P02751
488	3434-34_1	Fibronectin Fragment 3	P02751
489	3435-53_2	Fibronectin Fragment 4	P02751
490	3613-62_5	Ficolin-1	O00602
491	3313-21_2	Ficolin-2	Q15485
492	5462-62_3	Ficolin-3	O75636
493	3053-49_2	Fms-related tyrosine kinase 3 ligand	P49771
494	4986-59_1	Focal adhesion kinase 1	Q05397

495	3032-11_2	Follicle stimulating hormone	P01215, P01225
496	4132-27_2	Follistatin	P19883
497	13112-179_3	Follistatin-related protein 1	Q12841
498	3438-10_2	Follistatin-related protein 3	O95633
499	9213-24_3	Formimidoyltransferase-cyclodeaminase	O95954
500	2827-23_2	Fractalkine	P78423
501	5864-10_3	Fructose-bisphosphate aldolase A	P04075
502	5347-59_3	G2/mitotic-specific cyclin-B1	P14635
503	4548-4_2	Galactoside 3(4)-L-fucosyltransferase	P21217
504	11094-104_3	Galectin-10	Q05315
505	3033-57_1	Galectin-2	P05162
506	3066-12_1	Galectin-3	P17931
507	5000-52_1	Galectin-3-binding protein	Q08380
508	2982-82_2	Galectin-4	P56470
509	9196-8_3	Galectin-7	P47929
510	4909-68_1	Galectin-8	O00214
511	9197-4_3	Galectin-9	O00182
512	10339-48_3	Gamma-enolase	P09104
513	5897-58_3	Gastrin-releasing peptide	P07492
514	3314-74_2	GDNF family receptor alpha-1	P56159
515	2515-14_3	GDNF family receptor alpha-2	O00451
516	2505-49_3	GDNF family receptor alpha-3	O60609
517	4775-34_3	Gelsolin	P06396
518	3217-74_2	Glia-derived nexin	P07093
519	5822-22_3	Glial cell line-derived neurotrophic factor	P39905
520	3034-1_2	Glial fibrillary acidic protein	P14136
521	4891-50_1	Glucagon	P01275
522	2857-70_2	Glucocorticoid receptor	P04150
523	5223-59_3	Glucokinase regulatory protein	Q14397
524	4272-46_2	Glucose-6-phosphate isomerase	P06744
525	5478-50_2	Glutamate carboxypeptidase 2	Q04609
526	4993-16_1	Glutathione S-transferase A3	Q16772
527	4911-49_2	Glutathione S-transferase P	P09211
528	3848-14_2	Glyceraldehyde-3-phosphate dehydrogenase	P04406
529	11081-1_3	Glycerol-3-phosphate dehydrogenase [NAD(+)], cytoplasmic	P21695
530	3441-64_2	Glycogen synthase kinase-3 alpha/beta	P49840 P49841
531	5196-7_3	Glycylpeptide N-tetradecanoyltransferase 1	P30419
532	3315-15_1	Glypican-2	Q8N158
533	4842-62_2	Glypican-3	P51654
534	4991-12_1	Glypican-5	P78333
535	5350-14_2	Glypican-6	Q9Y625
536	4792-51_2	gp41 C34 peptide, HIV	Q70626
537	4992-49_1	Granulins	P28799
538	4840-73_1	Granulocyte colony-stimulating factor	P09919
539	2719-3_4	Granulocyte colony-stimulating factor receptor	Q99062
540	4697-59_2	Granulocyte-macrophage colony-stimulating factor	P04141
541	3195-50_2	Granulysin	P22749
542	3440-7_2	Granzyme A	P12544
543	4133-54_2	Granzyme B	P10144
544	3373-5_2	Granzyme H	P20718
545	5265-12_3	GRB2-related adapter protein 2	O75791
546	4449-67_3	Gremlin-1	O60565
547	2986-49_2	Gro-beta/gamma	P19876 P19875
548	2949-6_2	Group 10 secretory phospholipase A2	O15496
549	2447-7_4	Group IIE secretory phospholipase A2	Q9NZK7
550	5463-22_3	Growth arrest-specific protein 1	P54826

551	5464-52_3	Growth factor receptor-bound protein 2	P62993
552	2948-58_2	Growth hormone receptor	P10912
553	12060-28_3	Growth/differentiation factor 11	O95390
554	2765-4_3	Growth/differentiation factor 11/8	O95390 O14793
555	4374-45_2	Growth/differentiation factor 15	Q99988
556	4880-21_1	Growth/differentiation factor 2	Q9UK05
557	2752-62_2	Growth/differentiation factor 5	P43026
558	14583-49_3	Growth/differentiation factor 8	O14793
559	3067-67_1	Growth/differentiation factor 9	O60383
560	2985-35_1	Growth-regulated alpha protein	P09341
561	5193-51_3	GTPase KRas	P01116
562	4282-3_2	GTP-binding nuclear protein Ran	P62826
563	3054-3_2	Haptoglobin	P00738
564	4124-24_2	Heat shock 70 kDa protein 1A	P0DMV8
565	5903-91_2	Heat shock cognate 71 kDa protein	P11142
566	11103-24_3	Heat shock protein beta-1	P04792
567	2625-53_4	Heat shock protein HSP 90-alpha/beta	P07900 P08238
568	5467-15_3	Heat shock protein HSP 90-beta	P08238
569	2622-18_1	Heme oxygenase 2	P30519
570	11096-57_3	HemK methyltransferase family member 2	Q9Y5N5
571	4915-64_2	Hemoglobin	P69905, P68871
572	3332-57_1	Hemojuvelin	Q6ZVN8
573	2768-56_2	Hemopexin	P02790
574	5465-32_3	Heparan-sulfate 6-O-sulfotransferase 1	O60243
575	3316-58_1	Heparin cofactor 2	P05546
576	4134-4_2	Heparin-binding EGF-like growth factor	Q99075
577	5134-52_2	Hepatitis A virus cellular receptor 2	Q8TDQ0
578	2681-23_2	Hepatocyte growth factor	P14210
579	3617-80_4	Hepatocyte growth factor activator	Q04756
580	2837-3_2	Hepatocyte growth factor receptor	P08581
581	4407-10_1	Hepatocyte growth factor-like protein	P26927
582	4553-65_3	Hepatoma-derived growth factor-related protein 2	Q7Z4V5
583	3504-58_2	Hepcidin	P81172
584	14132-21_3	HERV-H LTR-associating protein 2	Q9UM44
585	4450-26_2	Heterogeneous nuclear ribonucleoprotein A/B	Q99729
586	4994-178_1	Heterogeneous nuclear ribonucleoprotein K	P61978
587	4224-7_2	Heterogeneous nuclear ribonucleoprotein Q	O60506
588	5351-52_3	Heterogeneous nuclear ribonucleoproteins A2/B1	P22626
589	13131-5_3	Hexokinase-1	P19367
590	13130-150_3	Hexokinase-2	P52789
591	5178-5_3	High affinity cAMP-specific 3',5'-cyclic phosphodiesterase 7A	Q13946
592	5201-50_4	High affinity cGMP-specific 3',5'-cyclic phosphodiesterase 9A	O76083
593	3312-64_1	High affinity immunoglobulin gamma Fc receptor I	P12314
594	3477-63_2	High affinity nerve growth factor receptor	P04629
595	2524-56_3	High mobility group protein B1	P09429
596	5900-11_2	Histidine triad nucleotide-binding protein 1	P49773
597	4996-66_1	Histidine-rich glycoprotein	P04196
598	2865-77_2	Histone acetyltransferase KAT6A	Q92794
599	2858-29_2	Histone acetyltransferase type B catalytic subunit	O14929
600	2859-69_2	Histone deacetylase 8	Q9BY41
601	2987-37_3	Histone H1.2	P16403
602	14144-3_3	Histone H2A type 3	Q7L7L0
603	4163-5_2	Histone H2A.z	P0C0S5
604	14143-8_3	Histone H2B type 2-E	Q16778
605	14146-92_3	Histone H3.1	P68431
606	5843-60_3	Histone-lysine N-methyltransferase EHMT2	Q96KQ7

607	2732-58_3	Homeobox protein NANOG	Q9H9S0
608	3443-61_2	Homeodomain-interacting protein kinase 3	Q9H422
609	3879-50_1	Hsp90 co-chaperone Cdc37	Q16543
610	4914-10_1	Human Chorionic Gonadotropin	P01215,P01233
611	3196-6_2	Hyaluronan and proteoglycan link protein 1	P10915
612	13089-6_3	Hypoxia-inducible factor 1-alpha	Q16665
613	5061-27_3	ICOS ligand	O75144
614	3198-4_1	Iduronate 2-sulfatase	P22304
615	11089-7_3	Immunoglobulin A	P01876 P01877
616	4987-17_1	Immunoglobulin alpha Fc receptor	P24071
617	4916-2_1	Immunoglobulin D	P01880
618	4135-84_2	Immunoglobulin E	P01854
619	2744-57_2	Immunoglobulin G	P01857
620	3069-52_3	Immunoglobulin M	P01871
621	13124-20_3	Immunoglobulin superfamily containing leucine-rich repeat protein 2	Q6UXK2
622	2860-19_2	Importin subunit alpha-1	P52292
623	3887-90_2	Importin subunit beta-1	Q14974
624	10358-33_3	Induced myeloid leukemia cell differentiation protein Mcl-1	Q07820
625	2988-57_2	Inducible T-cell costimulator	Q9Y6W8
626	2748-3_2	Inhibin beta A chain	P08476
627	8467-9_3	Inhibin beta A chain:Inhibin beta B chain heterodimer	P08476 P09529
628	3888-8_1	Inhibitor of growth protein 1	Q9UK53
629	5021-13_1	Inorganic pyrophosphatase	Q15181
630	5229-90_3	Inosine-5'-monophosphate dehydrogenase 1	P20839
631	5250-53_3	Inosine-5'-monophosphate dehydrogenase 2	P12268
632	4883-56_2	Insulin	P01308
633	3448-13_2	Insulin receptor	P06213
634	3197-70_2	Insulin-degrading enzyme	P14735
635	4232-19_2	Insulin-like growth factor 1 receptor	P08069
636	2952-75_2	Insulin-like growth factor I	P05019
637	2771-35_2	Insulin-like growth factor-binding protein 1	P08833
638	8469-41_3	Insulin-like growth factor-binding protein 2	P18065
639	2571-12_3	Insulin-like growth factor-binding protein 3	P17936
640	2950-57_2	Insulin-like growth factor-binding protein 4	P22692
641	2685-21_2	Insulin-like growth factor-binding protein 5	P24593
642	2686-67_2	Insulin-like growth factor-binding protein 6	P24592
643	3320-49_2	Insulin-like growth factor-binding protein 7	Q16270
644	3503-4_2	Integrin alpha-I: beta-1 complex	P56199, P05556
645	8488-33_3	Integrin alpha-IIb: beta-3 complex	P08514 P05106
646	4917-62_1	Integrin alpha-V: beta-5 complex	P06756, P18084
647	4811-33_3	Inter-alpha-trypsin inhibitor heavy chain H4	Q14624
648	4342-10_3	Intercellular adhesion molecule 1	P05362
649	5486-73_3	Intercellular adhesion molecule 2	P13598
650	2649-77_2	Intercellular adhesion molecule 3	P32942
651	5124-69_3	Intercellular adhesion molecule 5	Q9UMF0
652	9183-7_3	Interferon alpha/beta receptor 1	P17181
653	14128-121_3	Interferon alpha-10	P01566
654	3497-13_2	Interferon alpha-2	P01563
655	14129-1_3	Interferon alpha-7	P01567
656	14127-240_3	Interferon beta	P01574
657	14147-50_3	Interferon gamma	P01579
658	5825-49_3	Interferon gamma receptor 1	P15260
659	9180-6_3	Interferon gamma receptor 2	P38484
660	4396-54_1	Interferon lambda-1	Q8IU54
661	4397-26_2	Interferon lambda-2	Q8IZJ0
662	10351-51_3	Interferon regulatory factor 1	P10914

663	4851-25_1	Interleukin-1 alpha	P01583
664	3037-62_1	Interleukin-1 beta	P01584
665	2630-12_2	Interleukin-1 Receptor accessory protein	Q9NPH3
666	5353-89_2	Interleukin-1 receptor antagonist protein	P18510
667	2991-9_2	Interleukin-1 receptor type 1	P14778
668	14133-93_3	Interleukin-1 receptor type 2	P27930
669	4234-8_2	Interleukin-1 receptor-like 1	Q01638
670	2994-71_2	Interleukin-1 receptor-like 2	Q9HB29
671	2773-50_2	Interleukin-10	P22301
672	10344-334_3	Interleukin-10 receptor subunit alpha	Q13651
673	2631-50_2	Interleukin-10 receptor subunit beta	Q08334
674	4493-92_1	Interleukin-11	P20809
675	10367-62_3	Interleukin-12	P29459, P29460
676	2632-5_2	Interleukin-12 receptor subunit beta-1	P42701
677	3815-14_1	Interleukin-12 receptor subunit beta-2	Q99665
678	3072-4_2	Interleukin-13	P35225
679	2633-52_2	Interleukin-13 receptor subunit alpha-1	P78552
680	3445-53_2	Interleukin-15 receptor subunit alpha	Q13261
681	2774-10_3	Interleukin-16	Q14005
682	2992-59_2	Interleukin-17 receptor A	Q96F46
683	5084-154_3	Interleukin-17 receptor B	Q9NRM6
684	5468-67_3	Interleukin-17 receptor C	Q8NAC3
685	3376-49_2	Interleukin-17 receptor D	Q8NFM7
686	9170-24_3	Interleukin-17A	Q16552
687	3499-77_2	Interleukin-17B	Q9UHF5
688	4136-40_2	Interleukin-17D	Q8TAD2
689	2775-54_2	Interleukin-17F	Q96PD4
690	3446-7_2	Interleukin-18 receptor 1	Q13478
691	2993-1_2	Interleukin-18 receptor accessory protein	O95256
692	3073-51_2	Interleukin-18-binding protein	O95998
693	3035-80_2	Interleukin-19	Q9UHD0
694	3070-1_2	Interleukin-2	P60568
695	3151-6_1	Interleukin-2 receptor subunit alpha	P01589
696	4138-25_2	Interleukin-20	Q9NYY1
697	5085-18_3	Interleukin-20 receptor subunit alpha	Q9UHF4
698	2778-10_2	Interleukin-22	Q9GZX6
699	3620-67_2	Interleukin-22 receptor subunit alpha-1	Q8N6P7
700	5087-5_3	Interleukin-22 receptor subunit alpha-2	Q969J5
701	10365-132_3	Interleukin-23	P29460, Q9NPF7
702	5088-175_3	Interleukin-23 receptor	Q5VWK5
703	3321-2_2	Interleukin-24	Q13007
704	4137-57_2	Interleukin-25	Q9H293
705	2829-19_2	Interleukin-27	Q8NEV9 Q14213
706	5132-71_3	Interleukin-27 receptor subunit alpha	Q6UWB1
707	4717-55_2	Interleukin-3	P08700
708	4490-65_2	Interleukin-3 receptor subunit alpha	P26951
709	4556-10_2	Interleukin-34	Q6ZMJ4
710	14150-7_3	Interleukin-36 alpha	Q9UHA7
711	14149-9_3	Interleukin-36 beta	Q9NZH7
712	2723-9_2	Interleukin-37	Q9NZH6
713	2906-55_3	Interleukin-4	P05112
714	3055-54_2	Interleukin-4 receptor subunit alpha	P24394
715	11071-1_3	Interleukin-5	P05113
716	4491-4_2	Interleukin-5 receptor subunit alpha	Q01344
717	4673-13_2	Interleukin-6	P05231
718	4139-71_2	Interleukin-6 receptor subunit alpha	P08887

719	2620-4_2	Interleukin-6 receptor subunit beta	P40189
720	4140-3_2	Interleukin-7	P13232
721	5089-11_3	Interleukin-7 receptor subunit alpha	P16871
722	3447-64_2	Interleukin-8	P10145
723	5834-18_3	Interleukin-9	P15248
724	4924-32_1	Interstitial collagenase	P03956
725	2997-8_1	Junctional adhesion molecule B	P57087
726	2998-53_2	Junctional adhesion molecule C	Q9BX67
727	5094-62_3	Junctional adhesion molecule-like	Q86YT9
728	2831-29_1	Kallikrein-11	Q9UBX7
729	3199-54_2	Kallikrein-12	Q9UKR0
730	3200-49_2	Kallikrein-13	Q9UKR3
731	3681-87_3	Kallikrein-14	Q9P0G3
732	2833-20_1	Kallikrein-4	Q9Y5K2
733	3201-49_2	Kallikrein-5	Q9Y337
734	3450-4_2	Kallikrein-6	Q92876
735	3378-49_2	Kallikrein-7	P49862
736	2834-54_1	Kallikrein-8	O60259
737	3449-58_2	Kallistatin	P29622
738	8485-7_3	Kelch-like ECH-associated protein 1	Q14145
739	5354-11_3	Keratin, type I cytoskeletal 18	P05783
740	5095-21_3	Killer cell immunoglobulin-like receptor 2DL4	Q99706
741	5096-51_3	Killer cell immunoglobulin-like receptor 3DL2	P43630
742	5097-14_3	Killer cell immunoglobulin-like receptor 3DS1	Q14943
743	5098-79_3	Killer cell lectin-like receptor subfamily F member 1	Q9NZS2
744	4557-61_2	Kin of IRRE-like protein 3	Q8IZU9
745	5228-25_2	Kinesin-like protein KIF23	Q02241
746	4495-33_2	Kininogen-1	P01042
747	3202-28_2	Kremen protein 2	Q8NCW0
748	2828-82_2	Kunitz-type protease inhibitor 1	O43278
749	2843-13_2	Kunitz-type protease inhibitor 2	O43291
750	4559-64_2	Kynureninase	Q16719
751	4455-89_2	Lactadherin	Q08431
752	4801-13_3	Lactoperoxidase	P22079
753	2780-35_2	Lactotransferrin	P02788
754	3889-64_2	Lamin-B1	P20700
755	2728-62_2	Laminin	P25391, P07942, P11047
756	13133-73_3	Latent-transforming growth factor beta-binding protein 4	Q8N2S1
757	2635-61_2	Layilin	Q6UX15
758	3622-33_4	Legumain	Q99538
759	8484-24_3	Leptin	P41159
760	5400-52_3	Leptin receptor	P48357
761	4237-70_3	Leucine carboxyl methyltransferase 1	Q9UIC8
762	10990-21_3	Leucine-rich repeat serine/threonine-protein kinase 2	Q5S007
763	4452-9_2	Leucine-rich repeat transmembrane neuronal protein 1	Q86UE6
764	4453-83_3	Leucine-rich repeat transmembrane neuronal protein 3	Q86VH5
765	4547-59_2	Leucine-rich repeat transmembrane protein FLRT1	Q9NZU1
766	13122-19_3	Leucine-rich repeat transmembrane protein FLRT2	O43155
767	13123-3_3	Leucine-rich repeat transmembrane protein FLRT3	Q9NZU0
768	3322-52_2	Leucine-rich repeats and immunoglobulin-like domains protein 3	Q6UXM1
769	5837-49_3	Leukemia inhibitory factor receptor	P42702
770	5090-49_2	Leukocyte immunoglobulin-like receptor subfamily B member 1	Q8NHL6
771	5091-28_3	Leukocyte immunoglobulin-like receptor subfamily B member 2	Q8N423
772	6653-58_3	Leukocyte surface antigen CD47	Q08722
773	3204-2_2	Leukotriene A-4 hydrolase	P09960

774	4304-18_2	Ligand-dependent nuclear receptor corepressor-like protein	Q8N3X6
775	2999-6_2	Limbic system-associated membrane protein	Q13449
776	3074-6_2	Lipopolysaccharide-binding protein	P18428
777	13095-51_3	Lithostathine-1-alpha	P05451
778	3890-8_2	L-lactate dehydrogenase B chain	P07195
779	3291-30_2	Low affinity immunoglobulin epsilon Fc receptor	P06734
780	3309-2_2	Low affinity immunoglobulin gamma Fc region receptor II-a	P12318
781	3310-62_1	Low affinity immunoglobulin gamma Fc region receptor II-b	P31994
782	3311-27_1	Low affinity immunoglobulin gamma Fc region receptor III-B	O75015
783	3858-5_1	Low molecular weight phosphotyrosine protein phosphatase	P24666
784	13129-40_3	Low-density lipoprotein receptor	P01130
785	9182-3_3	Low-density lipoprotein receptor-related protein 1, soluble	Q07954
786	7640-29_3	Low-density lipoprotein receptor-related protein 1B	Q9NZR2
787	3323-37_1	Low-density lipoprotein receptor-related protein 8	Q14114
788	4831-4_2	L-Selectin	P14151
789	13114-50_3	Lumican	P51884
790	2953-31_2	Luteinizing hormone	P01215, P01229
791	13107-9_3	Ly6/PLAUR domain-containing protein 3	O95274
792	3206-4_2	Lymphatic vessel endothelial hyaluronic acid receptor 1	Q9Y5Y7
793	5099-14_3	Lymphocyte activation gene 3 protein	P18627
794	3623-84_4	Lymphocyte antigen 86	O95711
795	4143-74_2	Lymphotactin	P47992
796	3505-6_2	Lymphotoxin alpha1:beta2	P01374, Q06643
797	3506-49_1	Lymphotoxin alpha2:beta1	P01374, Q06643
798	4703-87_2	Lymphotoxin-alpha	P01374
799	3179-51_2	Lysosomal protective protein	P10619
800	5100-53_3	Lysosome membrane protein 2	Q14108
801	4920-10_1	Lysozyme C	P61626
802	3738-54_4	Macrophage colony-stimulating factor 1	P09603
803	2638-12_2	Macrophage colony-stimulating factor 1 receptor	P07333
804	2637-77_2	Macrophage mannose receptor 1	P22897
805	4496-60_2	Macrophage metalloelastase	P39900
806	5356-2_3	Macrophage migration inhibitory factor	P14174
807	3684-78_3	Macrophage scavenger receptor types I and II	P21757
808	4968-50_1	Macrophage-capping protein	P40121
809	2640-3_2	Macrophage-stimulating protein receptor	Q04912
810	3853-56_1	Malate dehydrogenase, cytoplasmic	P40925
811	5001-6_2	Mammaglobin-B	O75556
812	3605-77_4	Mannan-binding lectin serine protease 1	P48740
813	3000-66_1	Mannose-binding protein C	P11226
814	3820-68_2	MAP kinase-activated protein kinase 2	P49137
815	3822-54_2	MAP kinase-activated protein kinase 3	Q16644
816	3821-28_1	MAP kinase-activated protein kinase 5	Q8IW41
817	2475-1_3	Mast/stem cell growth factor receptor Kit	P10721
818	3325-2_2	Matrilin-2	O00339
819	3208-2_4	Matrilin-3	O15232
820	2789-26_2	Matrilysin	P09237
821	3209-69_1	Matrix extracellular phosphoglycoprotein	Q9NQ76
822	5002-76_1	Matrix metalloproteinase-14	P50281
823	5268-49_3	Matrix metalloproteinase-16	P51512
824	2838-53_1	Matrix metalloproteinase-17	Q9ULZ9
825	2579-17_5	Matrix metalloproteinase-9	P14780
826	5470-69_2	Mediator of RNA polymerase II transcription subunit 1	Q15648
827	3823-9_1	Megakaryocyte-associated tyrosine-protein kinase	P42679
828	2687-2_1	Melanoma-derived growth regulatory protein	Q16674
829	3685-53_1	Membrane frizzled-related protein	Q9BY79

830	3627-71_4	Membrane metallo-endopeptidase-like 1	Q495T6
831	3893-64_1	Mesothelin	Q13421
832	2211-9_6	Metalloproteinase inhibitor 1	P01033
833	2278-61_4	Metalloproteinase inhibitor 2	P16035
834	2480-58_3	Metalloproteinase inhibitor 3	P35625
835	3210-1_2	Methionine aminopeptidase 1	P53582
836	3170-6_1	Methionine aminopeptidase 2	P50579
837	3891-56_1	Methyl-CpG-binding domain protein 4	O95243
838	2730-58_2	MHC class I polypeptide-related sequence A	Q29983
839	5102-55_3	MHC class I polypeptide-related sequence B	Q29980
840	5854-60_3	Microtubule-associated protein tau	P10636
841	2911-27_2	Midkine	P21741
842	5280-68_5	Mitochondrial glutamate carrier 2	Q9H1K4
843	4545-53_3	Mitochondrial import inner membrane translocase subunit TIM14	Q96DA6
844	3115-64_2	Mitogen-activated protein kinase 1	P28482
845	5004-69_1	Mitogen-activated protein kinase 11	Q15759
846	5005-4_1	Mitogen-activated protein kinase 12	P53778
847	5006-71_1	Mitogen-activated protein kinase 13	O15264
848	5007-1_1	Mitogen-activated protein kinase 14	Q16539
849	2855-49_2	Mitogen-activated protein kinase 3	P27361
850	3825-18_2	Mitogen-activated protein kinase 8	P45983
851	5244-12_3	Mitogen-activated protein kinase 9	P45984
852	5259-2_3	Mitogen-activated protein kinase kinase kinase 7:TGF-beta-activated kinase 1 and MAP3K7-binding protein 1 fusion	O43318 Q15750
853	5009-11_1	Moesin	P26038
854	10364-6_3	Mothers against decapentaplegic homolog 2	Q15796
855	10363-13_3	Mothers against decapentaplegic homolog 3	P84022
856	9176-3_3	Mucin-1	P15941
857	4923-79_1	Muellerian-inhibiting factor	P03971
858	10362-35_3	Myc proto-oncogene protein	P01106
859	3514-49_2	Myeloblastin	P24158
860	3166-92_1	Myeloid cell surface antigen CD33	P20138
861	2580-83_2	Myeloperoxidase	P05164
862	3042-7_2	Myoglobin	P02144
863	7648-9_3	Myosin-binding protein C, slow-type	Q00872
864	3894-15_2	N-acetyl-D-glucosamine kinase	Q9UJ70
865	3616-3_5	N-acetylglucosamine-6-sulfatase	P15586
866	3173-49_2	N-acylethanolamine-hydrolyzing acid amidase	Q02083
867	5030-52_1	NAD-dependent protein deacetylase sirtuin-2	Q8IXJ6
868	2731-29_2	NADPH--cytochrome P450 reductase	P16435
869	3854-24_1	Nascent polypeptide-associated complex subunit alpha	Q13765
870	5104-57_3	Natural cytotoxicity triggering receptor 1	O76036
871	2734-49_4	Natural cytotoxicity triggering receptor 2	O95944
872	3003-29_2	Natural cytotoxicity triggering receptor 3	O14931
873	5063-12_2	Natural killer cell receptor 2B4	Q9BZW8
874	5139-32_3	Netrin receptor UNC5C	O95185
875	5140-56_3	Netrin receptor UNC5D	Q6UXZ4
876	6649-51_3	Netrin-1	O95631
877	3327-27_1	Netrin-4	Q9HB63
878	4498-62_2	Neural cell adhesion molecule 1, 120 kDa isoform	P13591
879	4246-40_2	Neural cell adhesion molecule L1	P32004
880	3601-54_3	Neural cell adhesion molecule L1-like protein	O00533
881	9178-30_3	Neuregulin-1	Q02297
882	14139-16_3	Neuregulin-4	Q8WWG1
883	5110-84_3	Neurexin-1-beta	P58400
884	5111-15_3	Neurexin-3-beta	Q9HDB5

885	4562-1_2	Neurexophilin-1	P58417
886	2944-66_2	Neuroblastoma suppressor of tumorigenicity 1	P41271
887	5107-7_2	Neurogenic locus notch homolog protein 1	P46531
888	5106-52_3	Neurogenic locus notch homolog protein 2	Q04721
889	5108-72_3	Neurogenic locus notch homolog protein 3	Q9UM47
890	5357-60_3	Neuroligin-4, X-linked	Q8N0W4
891	5109-24_3	Neuronal cell adhesion molecule	Q92823
892	13109-82_3	Neuronal growth regulator 1	Q7Z3B1
893	5542-22_2	Neuropilin-1	O14786
894	4145-58_2	Neurotrophin-3	P20783
895	4146-58_2	Neurotrophin-4	P34130
896	3212-30_3	Neutral ceramidase	Q9NR71
897	9172-69_3	Neutrophil collagenase	P22894
898	5308-89_2	Neutrophil elastase	P08246
899	2836-68_2	Neutrophil gelatinase-associated lipocalin	P80188
900	2790-54_2	Neutrophil-activating peptide 2	P02775
901	5011-11_1	Nicotinamide phosphoribosyltransferase	P43490
902	3213-65_2	Nidogen-1	P14543
903	3633-70_5	Nidogen-2	Q14112
904	3081-70_2	NKG2D ligand 1	Q9BZM6
905	3082-9_2	NKG2D ligand 2	Q9BZM5
906	2747-3_2	NKG2D ligand 3	Q9BZM4
907	3056-11_1	NKG2-D type II integral membrane protein	P26718
908	5846-24_3	Noggin	Q13253
909	9187-2_3	Non-histone chromosomal protein HMG-14	P05114
910	5260-80_3	Non-receptor tyrosine-protein kinase TYK2	P29597
911	4250-23_3	NSFL1 cofactor p47	Q9UNZ2
912	2658-27_1	NT-3 growth factor receptor	Q16288
913	7655-11_3	N-terminal pro-BNP	P16860
914	5236-2_3	Nuclear receptor subfamily 1 group D member 1	P20393
915	5909-51_3	Nucleoside diphosphate kinase A	P15531
916	4249-64_2	Nucleoside diphosphate kinase B	P22392
917	4254-6_2	NudC domain-containing protein 3	Q8IVD9
918	3635-76_4	OCIA domain-containing protein 1	Q9NX40
919	4769-10_3	Olfactomedin-4	Q6UX06
920	2693-20_3	Oncostatin-M	P13725
921	3634-5_4	Opioid-binding protein/cell adhesion molecule	Q14982
922	11067-13_3	Osteocalcin	P02818
923	5358-3_3	Osteomodulin	Q99983
924	13113-7_3	Osteopontin	P10451
925	5112-73_3	OX-2 membrane glycoprotein	P41217
926	3636-37_4	Oxidized low-density lipoprotein receptor 1	P78380
927	4588-1_2	Pancreatic hormone	P01298
928	4148-49_2	Pappalysin-1	Q13219
929	5954-62_3	Parathyroid hormone	P01270
930	2962-50_2	Parathyroid hormone-related protein	P12272
931	3727-35_1	Peptide YY	P10082
932	3329-14_2	Peptidoglycan recognition protein 1	O75594
933	3844-2_1	Peptidyl-prolyl cis-trans isomerase A	P62937
934	4718-5_2	Peptidyl-prolyl cis-trans isomerase B	P23284
935	5202-4_3	Peptidyl-prolyl cis-trans isomerase D	Q08752
936	5238-26_3	Peptidyl-prolyl cis-trans isomerase E	Q9UNP9
937	5248-68_2	Peptidyl-prolyl cis-trans isomerase F, mitochondrial	P30405
938	3457-57_1	Periostin	Q15063
939	3855-56_1	Peroxiredoxin-1	Q06830
940	5017-19_1	Peroxiredoxin-5, mitochondrial	P30044

941	5018-68_1	Peroxiredoxin-6	P30041
942	5915-58_3	Peroxisomal targeting signal 1 receptor	P50542
943	2696-87_2	Persephin	O60542
944	3847-56_2	Persulfide dioxygenase ETHE1, mitochondrial	O95571
945	4267-81_3	Pescadillo homolog	O00541
946	4276-10_2	Phosphatidylethanolamine-binding protein 1	P30086
947	3831-21_1	Phosphatidylinositol 3,4,5-trisphosphate 3-phosphatase and dual-specificity protein phosphatase PTEN	P60484
948	3391-10_2	Phosphatidylinositol 4,5-bisphosphate 3-kinase catalytic subunit gamma isoform	P48736
949	9173-21_3	Phosphoglucomutase-1	P36871
950	5020-50_1	Phosphoglycerate kinase 1	P00558
951	3896-5_2	Phosphoglycerate mutase 1	P18669
952	4910-21_1	Phospholipase A2	P04054
953	2692-74_2	Phospholipase A2, membrane associated	P14555
954	9211-19_3	Pigment epithelium-derived factor	P36955
955	3390-72_2	PIK3CA/PIK3R1	P42336 P27986
956	14114-18_3	PILR alpha-associated neural protein	Q8IYJ0
957	8446-4_3	Pituitary adenylate cyclase-activating polypeptide 27	P18509
958	8450-36_3	Pituitary adenylate cyclase-activating polypeptide 38	P18509
959	3078-1_2	Placenta growth factor	P49763
960	4152-58_2	Plasma kallikrein	P03952
961	4479-14_2	Plasma protease C1 inhibitor	P05155
962	3389-7_2	Plasma serine protease inhibitor	P05154
963	4150-75_2	Plasmin	P00747
964	4151-6_2	Plasminogen	P00747
965	2925-9_1	Plasminogen activator inhibitor 1	P05121
966	2695-25_1	Platelet endothelial cell adhesion molecule	P16284
967	2697-7_2	Platelet factor 4	P02776
968	2973-15_2	Platelet glycoprotein 4	P16671
969	4990-87_1	Platelet glycoprotein Ib alpha chain	P07359
970	3194-36_2	Platelet glycoprotein VI	Q9HCN6
971	14123-34_3	Platelet receptor Gi24	Q9H7M9
972	5015-15_1	Platelet-activating factor acetylhydrolase	Q13093
973	2642-4_1	Platelet-activating factor acetylhydrolase IB subunit beta	P68402
974	3828-54_2	Platelet-derived growth factor C	Q9NRA1
975	10366-11_3	Platelet-derived growth factor receptor alpha	P16234
976	3459-49_2	Platelet-derived growth factor receptor beta	P09619
977	4499-21_1	Platelet-derived growth factor subunit A	P04085
978	4149-8_2	Platelet-derived growth factor subunit B	P01127
979	3045-72_2	Pleiotrophin	P21246
980	9216-100_3	Plexin-B2	O15031
981	4564-2_2	Plexin-C1	O60486
982	3216-2_2	Polymeric immunoglobulin receptor	P01833
983	6641-60_3	PolyUbiquitin K48-linked	P0CG47
984	6647-55_3	PolyUbiquitin K63-linked	P0CG48
985	4271-75_2	Prefoldin subunit 5	Q99471
986	5060-62_3	Programmed cell death 1 ligand 1	Q9NZQ7
987	3004-67_2	Programmed cell death 1 ligand 2	Q9BQ51
988	2247-20_11	Prokineticin-1	P58294
989	2585-2_5	Prolactin	P01236
990	5114-65_3	Prolactin receptor	P16471
991	2915-6_2	Proliferating cell nuclear antigen	P12004
992	4258-15_2	Proliferation-associated protein 2G4	Q9UQ80
993	5029-3_1	Prolyl endopeptidase FAP	Q12884
994	9204-33_3	Pro-opiomelanocortin	P01189
995	2960-66_2	Properdin	P27918

996	4459-68_2	Proprotein convertase subtilisin/kexin type 7	Q16549
997	5231-79_3	Proprotein convertase subtilisin/kexin type 9	Q8NBP7
998	3761-4_2	Prostaglandin G/H synthase 2	P35354
999	8468-19_3	Prostate-specific antigen	P07288
1000	5918-5_3	Proteasome activator complex subunit 1	Q06323
1001	5204-13_3	Proteasome activator complex subunit 3	P61289
1002	3859-50_2	Proteasome subunit alpha type-1	P25786
1003	4280-47_2	Proteasome subunit alpha type-2	P25787
1004	3860-7_2	Proteasome subunit alpha type-6	P60900
1005	4706-17_2	Protein 4.1	P11171
1006	4322-28_3	Protein amnionless	Q9BXJ7
1007	5016-61_2	Protein deglycase DJ-1	Q99497
1008	4278-14_3	Protein disulfide-isomerase	P07237
1009	4719-58_2	Protein disulfide-isomerase A3	P30101
1010	2623-54_4	Protein E7_HPV16	P03129
1011	2624-31_2	Protein E7_HPV18	P06788
1012	2760-2_2	Protein FAM107A	O95990
1013	4774-62_3	Protein FAM107B	Q9H098
1014	9177-6_3	Protein FAM3B	P58499
1015	13102-1_3	Protein FAM3D	Q96BQ1
1016	5092-51_3	Protein jagged-1	P78504
1017	5093-47_3	Protein jagged-2	Q9Y219
1018	2644-11_2	Protein kinase C alpha type	P17252
1019	5475-10_3	Protein kinase C beta type (splice variant beta-II)	P05771
1020	2869-68_3	Protein kinase C delta type	Q05655
1021	5476-66_3	Protein kinase C gamma type	P05129
1022	3379-29_1	Protein kinase C iota type	P41743
1023	3817-18_1	Protein kinase C theta type	Q04759
1024	2645-54_1	Protein kinase C zeta type	Q05513
1025	4238-4_2	Protein lin-7 homolog B	Q9HAP6
1026	2737-22_2	Protein NOV homolog	P48745
1027	2769-3_2	Protein Rev_HV2BE	P18093
1028	5852-6_3	Protein S100-A12	P80511
1029	14116-129_3	Protein S100-A4	P26447
1030	13090-17_3	Protein S100-A6	P06703
1031	5921-58_3	Protein S100-A7	P31151
1032	5339-49_3	Protein S100-A9	P06702
1033	5364-7_3	Protein SET	Q01105
1034	4889-82_1	Protein Wnt-7a	O00755
1035	13119-26_3	Protein Z-dependent protease inhibitor	Q9UK55
1036	4471-50_2	Protein-glutamine gamma-glutamyltransferase E	Q08188
1037	3832-51_1	Protein-tyrosine kinase 6	Q13882
1038	5316-54_3	Prothrombin	P00734
1039	3220-40_2	Proto-oncogene tyrosine-protein kinase receptor Ret	P07949
1040	5488-74_3	Proto-oncogene tyrosine-protein kinase Src	P12931
1041	5275-28_2	Proto-oncogene vav	P15498
1042	4154-57_2	P-selectin	P16109
1043	4414-69_2	Pulmonary surfactant-associated protein D	P35247
1044	11098-1_3	Pyridoxal kinase	O00764
1045	3897-61_1	Pyridoxal phosphate phosphatase	Q96GD0
1046	4240-31_2	Pyruvate kinase PKM	P14618
1047	9207-60_3	Quinone oxidoreductase-like protein 1	O95825
1048	2647-66_2	Rab GDP dissociation inhibitor beta	P50395
1049	3392-68_2	RAC-alpha/beta/gamma serine/threonine-protein kinase	P31749 P31751 Q9Y243
1050	5360-9_2	RAC-beta serine/threonine-protein kinase	P31751

1051	5481-16_3	Ras GTPase-activating protein 1	P20936
1052	2870-29_2	Ras-related C3 botulinum toxin substrate 1	P63000
1053	5271-5_1	Ras-related C3 botulinum toxin substrate 3	P60763
1054	2616-23_18	Receptor tyrosine-protein kinase erbB-2	P04626
1055	2617-56_35	Receptor tyrosine-protein kinase erbB-3	P21860
1056	2618-10_2	Receptor tyrosine-protein kinase erbB-4	Q15303
1057	3437-80_3	Receptor-type tyrosine-protein kinase FLT3	P36888
1058	11102-22_3	Regenerating islet-derived protein 4	Q9BYZ8
1059	14135-3_3	Relaxin receptor 1	Q9HBX9
1060	3396-54_2	Renin	P00797
1061	5483-1_3	Repulsive guidance molecule A	Q96B86
1062	3046-31_1	Resistin	Q9HD89
1063	5118-74_2	Reticulon-4	Q9NQC3
1064	5105-2_3	Reticulon-4 receptor	Q9BZR6
1065	5024-67_1	Retinoblastoma-associated protein	P06400
1066	3079-62_2	Retinoic acid receptor responder protein 2	Q99969
1067	5482-61_3	Retinol-binding protein 4	P02753
1068	3331-8_1	RGM domain family member B	Q6NW40
1069	8470-213_3	Ribonuclease H1	O60930
1070	3469-74_1	Ribosomal protein S6 kinase alpha-3	P51812
1071	3865-53_1	Ribosomal protein S6 kinase alpha-5	O75582
1072	3866-7_2	Ribosome maturation protein SBDS	Q9Y3A5
1073	4284-18_3	RNA-binding protein 39	Q14498
1074	5116-62_2	Roundabout homolog 2	Q9HCK4
1075	5117-14_3	Roundabout homolog 3	Q96MS0
1076	4566-24_3	R-spondin-2	Q6UXX9
1077	13094-75_3	R-spondin-3	Q9BXY4
1078	8464-31_3	R-spondin-4	Q2I0M5
1079	5129-12_3	Scavenger receptor class F member 1	Q14162
1080	5130-67_3	Scavenger receptor class F member 2	Q96GP6
1081	5028-59_1	Scavenger receptor cysteine-rich type 1 protein M130	Q86VB7
1082	13101-60_3	Sclerostin	Q9BQB4
1083	13093-6_3	Secreted and transmembrane protein 1	Q8WVN6
1084	3221-54_1	Secreted frizzled-related protein 1	Q8N474
1085	2841-13_2	Secreted frizzled-related protein 3	Q92765
1086	3728-52_2	Secretin	P09683
1087	3867-49_1	Seizure 6-like protein 2	Q6UXD5
1088	3222-11_2	Semaphorin-3A	Q14563
1089	5363-51_3	Semaphorin-3E	O15041
1090	13132-14_3	Semaphorin-5A	Q13591
1091	5122-92_2	Semaphorin-6A	Q9H2E6
1092	5121-3_2	Semaphorin-6B	Q9H3T3
1093	3624-3_4	Serine protease 27	Q9BQR3
1094	3317-33_1	Serine protease HTRA2, mitochondrial	O43464
1095	3471-49_1	Serine/threonine-protein kinase 16	O75716
1096	5249-31_3	Serine/threonine-protein kinase 17B	O94768
1097	2853-68_2	Serine/threonine-protein kinase Chk1	O14757
1098	3360-50_4	Serine/threonine-protein kinase Chk2	O96017
1099	3629-60_4	Serine/threonine-protein kinase MRCK beta	Q9Y5S2
1100	3387-1_2	Serine/threonine-protein kinase PAK 3	O75914
1101	3827-22_1	Serine/threonine-protein kinase PAK 6	Q9NQU5
1102	3388-58_2	Serine/threonine-protein kinase PAK 7	Q9P286
1103	5359-65_3	Serine/threonine-protein kinase pim-1	P11309
1104	3394-81_2	Serine/threonine-protein kinase PLK1	P53350
1105	2969-11_1	Serine/threonine-protein kinase receptor R3	P37023
1106	3400-49_2	Serine/threonine-protein kinase TBK1	Q9UHD2

1107	5493-17_3	Serine/threonine-protein kinase WNK3	Q9BYP7
1108	4162-54_2	Serotransferrin	P02787
1109	3707-12_2	Serum albumin	P02768
1110	4336-2_1	Serum amyloid A-1 protein	P0DJI8
1111	2474-54_5	Serum amyloid P-component	P02743
1112	4261-55_2	Serum paraoxonase/arylesterase 1	P27169
1113	4929-55_1	Sex hormone-binding globulin	P04278
1114	4984-83_1	S-formylglutathione hydrolase	P10768
1115	4567-82_2	SH2 domain-containing protein 1A	O60880
1116	5272-55_2	SHC-transforming protein 1	P29353
1117	5125-6_3	Sialic acid-binding Ig-like lectin 14	Q08ET2
1118	2741-22_2	Sialic acid-binding Ig-like lectin 6	O43699
1119	2742-68_2	Sialic acid-binding Ig-like lectin 7	Q9Y286
1120	3007-7_2	Sialic acid-binding Ig-like lectin 9	Q9Y336
1121	4464-10_2	Sialoadhesin	Q9BZZ2
1122	10370-21_3	Signal transducer and activator of transcription 1-alpha/beta	P42224
1123	10346-5_3	Signal transducer and activator of transcription 3	P40763
1124	10372-18_3	Signal transducer and activator of transcription 6	P42226
1125	3642-4_1	SLAM family member 5	Q9UIB8
1126	5128-53_3	SLAM family member 6	Q96DU3
1127	5487-7_3	SLAM family member 7	Q9NQ25
1128	3643-90_2	SLIT and NTRK-like protein 1	Q96PX8
1129	4568-17_2	SLIT and NTRK-like protein 5	O94991
1130	3868-8_1	Small glutamine-rich tetratricopeptide repeat-containing protein alpha	O43765
1131	5494-52_3	Small nuclear ribonucleoprotein F	P62306
1132	9169-14_3	Small ubiquitin-related modifier 3	P55854
1133	5957-30_3	Somatostatin-28	P61278
1134	8462-18_3	Somatotropin	P01241
1135	2743-5_2	Sonic hedgehog protein	Q15465
1136	3903-49_2	Sorting nexin-4	O95219
1137	3043-49_2	SPARC	P09486
1138	4467-49_2	SPARC-like protein 1	Q14515
1139	13118-5_3	SPARC-related modular calcium-binding protein 1	Q9H4F8
1140	5031-10_1	Spectrin alpha chain, non-erythrocytic 1	Q13813
1141	3902-21_2	S-phase kinase-associated protein 1	P63208
1142	4294-16_2	Sphingosine kinase 1	Q9NYA1
1143	4468-21_2	Sphingosine kinase 2	Q9NRA0
1144	4297-62_3	Spondin-1	Q9HCB6
1145	3399-31_2	Stabilin-2	Q8WWQ8
1146	4930-21_1	Stanniocalcin-1	P52823
1147	4500-50_2	Stem cell growth factor-alpha	Q9Y240
1148	2966-65_2	Stem Cell Growth Factor-beta	Q9Y240
1149	5489-18_3	Stress-induced-phosphoprotein 1	P31948
1150	3516-60_2	Stromal cell-derived factor 1	P48061
1151	2788-55_1	Stromelysin-1	P08254
1152	8479-4_3	Stromelysin-2	P09238
1153	2877-3_1	SUMO-conjugating enzyme UBC9	P63279
1154	2794-60_2	Superoxide dismutase [Cu-Zn]	P00441
1155	5008-51_1	Superoxide dismutase [Mn], mitochondrial	P04179
1156	13105-7_3	Synaptosomal-associated protein 25	P60880
1157	3766-51_4	Syntaxin-1A	Q16623
1158	3232-28_2	Tartrate-resistant acid phosphatase type 5	P13686
1159	2875-15_2	TATA-box-binding protein	P20226
1160	3143-3_1	T-cell surface glycoprotein CD4	P01730
1161	4155-3_2	Tenascin	P24821
1162	5810-25_3	Teratocarcinoma-derived growth factor 1	P13385

1163	5490-53_3	Testican-1	Q08629
1164	5491-12_3	Testican-2	Q92563
1165	5133-17_3	TGF-beta receptor type-2	P37173
1166	4815-25_3	Thioredoxin domain-containing protein 12	O95881
1167	4157-2_1	Thrombin	P00734
1168	5947-90_3	Thrombopoietin	P40225
1169	3473-78_2	Thrombopoietin Receptor	P40238
1170	3474-19_2	Thrombospondin-1	P07996
1171	3339-33_1	Thrombospondin-2	P35442
1172	3340-53_1	Thrombospondin-4	P35443
1173	3010-53_2	Thymic stromal lymphopoietin	Q969D9
1174	4301-58_2	Thymidine kinase, cytosolic	P04183
1175	5035-7_1	Thymidylate synthase	P04818
1176	4165-2_1	Thyroglobulin	P01266
1177	3873-51_2	Thyroid peroxidase	P07202
1178	3521-16_2	Thyroid Stimulating Hormone	P01215 P01222
1179	2706-69_2	Thyroxine-binding globulin	P05543
1180	4931-59_1	Tissue Factor	P13726
1181	3336-50_1	Tissue factor pathway inhibitor	P10646
1182	2212-69_1	Tissue-type plasminogen activator	P00750
1183	2603-61_2	T-lymphocyte activation antigen CD80	P33681
1184	5337-64_3	T-lymphocyte activation antigen CD86	P42081
1185	3324-51_1	T-lymphocyte surface antigen Ly-9	Q9HBG7
1186	3835-11_2	Toll-like receptor 2	O60603
1187	11101-18_3	Toll-like receptor 4	O00206
1188	3647-49_4	Toll-like receptor 4:Lymphocyte antigen 96 complex	O00206 Q9Y6Y9
1189	10356-21_3	Transcription factor AP-1	P05412
1190	5384-67_3	Transcription factor IIIB 90 kDa subunit	Q92994
1191	3009-3_2	Transforming growth factor beta receptor type 3	Q03167
1192	2333-72_1	Transforming growth factor beta-1	P01137
1193	4156-74_1	Transforming growth factor beta-2	P61812
1194	3520-58_1	Transforming growth factor beta-3	P10600
1195	3283-21_1	Transforming growth factor-beta-induced protein ig-h3	Q15582
1196	9201-13_3	Transgelin-2	P37802
1197	4306-4_2	Transketolase	P29401
1198	3872-2_1	Translationally-controlled tumor protein	P13693
1199	5080-131_3	Transmembrane glycoprotein NMB	Q14956
1200	9185-15_3	Trefoil factor 1	P04155
1201	9191-8_3	Trefoil factor 2	Q03403
1202	4721-54_2	Trefoil factor 3	Q07654
1203	4309-59_3	Triosephosphate isomerase	P60174
1204	5033-27_1	Tropomyosin alpha-1 chain	P09493
1205	7660-21_3	Tropomyosin alpha-4 chain	P67936
1206	4472-5_2	Tropomyosin beta chain	P07951
1207	5441-67_3	Troponin I, cardiac muscle	P19429
1208	5440-26_3	Troponin I, fast skeletal muscle	P48788
1209	5315-22_3	Troponin T, cardiac muscle	P45379
1210	3049-61_2	Trypsin-1	P07477
1211	5034-79_1	Trypsin-2	P07478
1212	3479-71_2	Trypsin-3	P35030
1213	3403-1_2	Tryptase beta-2	P20231
1214	3404-51_2	Tryptase gamma	Q9NRR2
1215	5936-53_3	Tumor necrosis factor	P01375
1216	2917-3_2	Tumor necrosis factor ligand superfamily member 11	O14788
1217	5939-42_3	Tumor necrosis factor ligand superfamily member 12	O43508
1218	3059-50_2	Tumor necrosis factor ligand superfamily member 13B	Q9Y275

1219	5355-69_3	Tumor necrosis factor ligand superfamily member 14	O43557
1220	2968-61_1	Tumor necrosis factor ligand superfamily member 15	O95150
1221	2708-54_2	Tumor necrosis factor ligand superfamily member 18	Q9UNG2
1222	2839-2_1	Tumor necrosis factor ligand superfamily member 4	P23510
1223	3052-8_2	Tumor necrosis factor ligand superfamily member 6, soluble form	P48023
1224	3421-54_2	Tumor necrosis factor ligand superfamily member 8	P32971
1225	2599-51_2	Tumor necrosis factor ligand superfamily member 9	P41273
1226	4832-75_2	Tumor necrosis factor receptor superfamily member 10A	O00220
1227	5534-49_2	Tumor necrosis factor receptor superfamily member 10B	O14763
1228	14121-24_3	Tumor necrosis factor receptor superfamily member 10D	Q9UBN6
1229	5424-55_3	Tumor necrosis factor receptor superfamily member 11A	Q9Y6Q6
1230	2526-53_3	Tumor necrosis factor receptor superfamily member 11B	O00300
1231	5138-50_3	Tumor necrosis factor receptor superfamily member 12A	Q9NP84
1232	2704-74_1	Tumor necrosis factor receptor superfamily member 13B	O14836
1233	5383-14_3	Tumor necrosis factor receptor superfamily member 13C	Q96RJ3
1234	5352-11_3	Tumor necrosis factor receptor superfamily member 14	Q92956
1235	2665-26_2	Tumor necrosis factor receptor superfamily member 17	Q02223
1236	5526-53_3	Tumor necrosis factor receptor superfamily member 18	Q9Y5U5
1237	5131-15_3	Tumor necrosis factor receptor superfamily member 19	Q9NS68
1238	5115-31_3	Tumor necrosis factor receptor superfamily member 19L	Q969Z4
1239	2654-19_1	Tumor necrosis factor receptor superfamily member 1A	P19438
1240	3152-57_1	Tumor necrosis factor receptor superfamily member 1B	P20333
1241	5404-53_3	Tumor necrosis factor receptor superfamily member 21	O75509
1242	5073-30_2	Tumor necrosis factor receptor superfamily member 25	Q93038
1243	3083-71_1	Tumor necrosis factor receptor superfamily member 27	Q9HAV5
1244	2636-10_2	Tumor necrosis factor receptor superfamily member 3	P36941
1245	3730-81_2	Tumor necrosis factor receptor superfamily member 4	P43489
1246	5392-73_2	Tumor necrosis factor receptor superfamily member 6	P25445
1247	5070-76_3	Tumor necrosis factor receptor superfamily member 6B	O95407
1248	2605-49_2	Tumor necrosis factor receptor superfamily member 8	P28908
1249	2598-9_3	Tumor necrosis factor receptor superfamily member 9	Q07011
1250	2977-7_2	Tumor necrosis factor receptor superfamily member EDAR	Q9UNE0
1251	5036-50_1	Tumor necrosis factor-inducible gene 6 protein	P98066
1252	2619-72_2	Tumor-associated calcium signal transducer 2	P09758
1253	3341-33_4	Tyrosine-protein kinase ABL1	P00519
1254	3416-2_2	Tyrosine-protein kinase BTK	Q06187
1255	3363-31_4	Tyrosine-protein kinase CSK	P41240
1256	4220-39_2	Tyrosine-protein kinase Fer	P16591
1257	3810-50_2	Tyrosine-protein kinase Fgr	P09769
1258	3813-3_2	Tyrosine-protein kinase Fyn	P06241
1259	3374-49_2	Tyrosine-protein kinase HCK	P08631
1260	4998-50_2	Tyrosine-protein kinase JAK2	O60674
1261	3452-17_2	Tyrosine-protein kinase Lck	P06239
1262	3453-87_2	Tyrosine-protein kinase Lyn	P07948
1263	3381-24_2	Tyrosine-protein kinase Lyn, isoform B	P07948
1264	2844-53_2	Tyrosine-protein kinase receptor Tie-1, soluble	P35590
1265	2611-72_2	Tyrosine-protein kinase receptor TYRO3	Q06418
1266	3646-7_4	Tyrosine-protein kinase Tec	P42680
1267	8474-6_3	Tyrosine-protein kinase transmembrane receptor ROR1	Q01973
1268	2878-66_2	Tyrosine-protein kinase Yes	P07947
1269	4476-22_2	Tyrosine-protein kinase ZAP-70	P43403
1270	3005-5_2	Tyrosine-protein phosphatase non-receptor type 1	P18031
1271	3397-7_4	Tyrosine-protein phosphatase non-receptor type 11	Q06124
1272	3401-8_2	Tyrosine-protein phosphatase non-receptor type 2	P17706
1273	4318-12_3	Tyrosine-protein phosphatase non-receptor type 6	P29350
1274	5430-66_3	Tyrosine-protein phosphatase non-receptor type substrate 1	P78324

1275	4474-19_2	Ubiquitin	P62979
1276	9215-117_3	Ubiquitin carboxyl-terminal hydrolase 25	Q9UHP3
1277	5019-16_2	Ubiquitin carboxyl-terminal hydrolase isozyme L1	P09936
1278	2846-24_2	Ubiquitin+1, truncated mutation for UbB	P62979
1279	9199-6_3	Ubiquitin-conjugating enzyme E2 G2	P60604
1280	3874-8_1	Ubiquitin-conjugating enzyme E2 L3	P68036
1281	3905-62_1	Ubiquitin-conjugating enzyme E2 N	P61088
1282	3836-51_2	Ubiquitin-fold modifier 1	P61960
1283	3405-6_2	Ubiquitin-fold modifier-conjugating enzyme 1	Q9Y3C8
1284	14151-4_3	Ubiquitin-like protein ISG15	P05161
1285	4786-58_3	UMP-CMP kinase	P30085
1286	2652-15_1	Urokinase plasminogen activator surface receptor	Q03405
1287	4158-54_2	Urokinase-type plasminogen activator	P00749
1288	4209-60_2	Vacuolar protein sorting-associated protein VTA1 homolog	Q9NP79
1289	2967-8_1	Vascular cell adhesion protein 1	P19320
1290	2597-8_3	Vascular endothelial growth factor A	P15692
1291	4867-15_2	Vascular endothelial growth factor A, isoform 121	P15692
1292	3132-1_1	Vascular endothelial growth factor C	P49767
1293	13098-93_3	Vascular endothelial growth factor D	O43915
1294	3651-50_5	Vascular endothelial growth factor receptor 2	P35968
1295	2358-19_2	Vascular endothelial growth factor receptor 3	P35916
1296	3522-57_1	Vasoactive Intestinal Peptide	P01282
1297	7638-30_3	Vesicular integral-membrane protein VIP36	Q12907
1298	2961-1_2	Vitamin K-dependent protein C	P04070
1299	2700-56_2	Vitamin K-dependent protein S	P07225
1300	13125-45_3	Vitronectin	P04004
1301	3050-7_2	von Willebrand factor	P04275
1302	4569-52_2	VPS10 domain-containing receptor SorCS2	Q96PQ0
1303	3191-50_2	WAP, kazal, immunoglobulin, kunitz and NTR domain-containing protein 1	Q96NZ8
1304	3235-50_2	WAP, Kazal, immunoglobulin, Kunitz and NTR domain-containing protein 2	Q8TEU8
1305	2848-2_2	Wnt inhibitory factor 1	Q9Y5W5
1306	3057-55_1	WNT1-inducible-signaling pathway protein 1	O95388
1307	5927-4_3	WNT1-inducible-signaling pathway protein 3	O95389
1308	3481-87_1	Xaa-Pro aminopeptidase 1	Q9NQW7
1309	5082-51_3	X-linked interleukin-1 receptor accessory protein-like 2	Q9NP60
1310	2835-1_4	X-ray repair cross-complementing protein 6	P12956
